# Short-term excess mortality following tropical cyclones in the United States

**DOI:** 10.1126/sciadv.adg6633

**Published:** 2023-08-16

**Authors:** Robbie M. Parks, Vasilis Kontis, G. Brooke Anderson, Jane W. Baldwin, Goodarz Danaei, Ralf Toumi, Francesca Dominici, Majid Ezzati, Marianthi-Anna Kioumourtzoglou

**Affiliations:** ^1^ Department of Environmental Health Sciences, Mailman School of Public Health, Columbia University, New York, NY, USA.; ^2^ MRC Centre for Environment and Health, School of Public Health, Imperial College London, London, UK.; ^3^ Department of Environmental and Radiological Health Sciences, Colorado State University, Fort Collins, CO, USA.; ^4^ Department of Earth System Science, University of California, Irvine, Irvine, CA, USA.; ^5^ Lamont-Doherty Earth Observatory, Palisades, NY, USA.; ^6^ Department of Global Health and Population, T. H. Chan School of Public Health, Harvard University, Boston, MA, USA.; ^7^ Space and Atmospheric Physics Imperial College London, London, UK.; ^8^ Department of Biostatistics, T. H. Chan School of Public Health, Harvard University, Boston, MA, USA.; ^9^ Abdul Latif Jameel Institute for Disease and Emergency Analytics, Imperial College London, London, UK.

## Abstract

Knowledge of excess deaths after tropical cyclones is critical to understanding their impacts, directly relevant to policies on preparedness and mitigation. We applied an ensemble of 16 Bayesian models to 40.7 million U.S. deaths and a comprehensive record of 179 tropical cyclones over 32 years (1988–2019) to estimate short-term all-cause excess deaths. The deadliest tropical cyclone was Hurricane Katrina in 2005, with 1491 [95% credible interval (CrI): 563, 3206] excess deaths (>99% posterior probability of excess deaths), including 719 [95% CrI: 685, 752] in Orleans Parish, LA (>99% probability). Where posterior probabilities of excess deaths were >95%, there were 3112 [95% CrI: 2451, 3699] total post–hurricane force excess deaths and 15,590 [95% CrI: 12,084, 18,835] post–gale to violent storm force deaths; 83.1% of post–hurricane force and 70.0% of post–gale to violent storm force excess deaths occurred more recently (2004–2019); and 6.2% were in least socially vulnerable counties.

## Teaser

U.S. post-tropical cyclone excess deaths were greatest recently (2004-2019) and for the most socially vulnerable communities.

## INTRODUCTION

Tropical cyclones have a devastating impact on society throughout many parts of the world (*1*–*3*). Trends of heightened activity and increased intensity of tropical cyclones in recent years indicate that tropical cyclone exposure is and will remain a major public health concern (*4*, *5*). In the aftermath of a tropical cyclone, deaths can result from several major causes, including injuries, infectious and parasitic diseases, cardiovascular diseases, neuropsychiatric conditions, and respiratory diseases (*5*). In the United States, states in the Atlantic and Gulf Coasts, most frequently exposed to tropical cyclones, contain nearly half the population of the entire country, and their populations continue to grow fast. Some of the wealthiest and poorest communities in the United States are located in tropical cyclone–affected areas. Although tropical cyclones are not selective of communities they may affect, the impact on public health depends on community resilience.

Knowledge of short-term excess deaths, i.e., the difference between the observed number of deaths in the immediate aftermath post–tropical cyclone and the counterfactual number of deaths had a cyclone not occurred, is essential for understanding the public health burden of climate-related disasters, directly relevant to policies on preparedness and mitigation and a key recommended measure for post-disaster mortality assessment (*6*, *7*). The spatial variation of excess mortality after a single catastrophic extreme event, such as a tropical cyclone, also informs how vulnerability permeates society through long-term institutional neglect; excess mortality is not merely a product of the hazards of a tropical cyclone but rather a combination of environmental and social factors (*8*, *9*). However, the methodology to attribute mortality to tropical cyclones has been hitherto inconsistent. Tropical cyclone death counts are, therefore, not typically directly comparable with each other; even estimates of the same tropical cyclone can vary greatly, such as Hurricane Maria in 2017, for which official death counts were up to 70 times lower than the total number of excess deaths (*10*).

Here, we used death registration data across more than three decades to directly estimate the number of excess deaths after tropical cyclones in all affected areas in the United States (*11*). We present estimates by county, year, tropical cyclone name, and strength of tropical cyclone, the variation of which from year to year implicitly accounts for how active a tropical cyclone season was, showing where the death toll after tropical cyclones was largest. We also investigated how excess mortality after tropical cyclones varied by social vulnerability.

## RESULTS

**Fig. 1.**
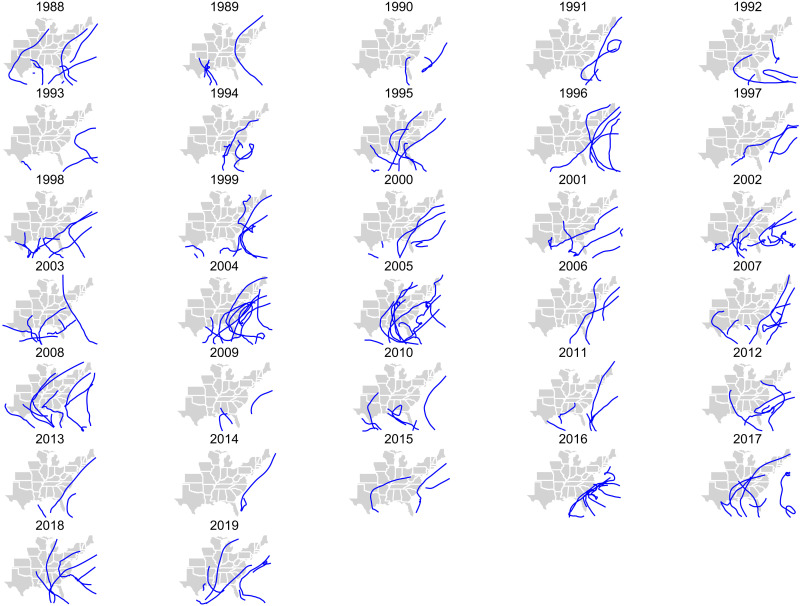
Best-track routes of each tropical cyclone by year, 1988–2019.

**Fig. 2.**
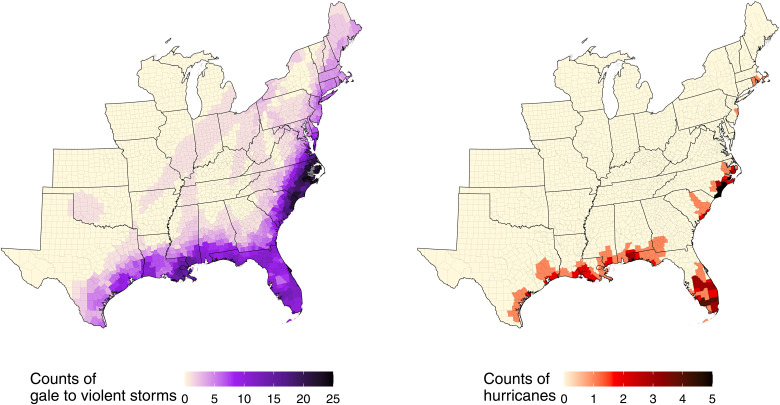
Counts of tropical cyclones categorized by gale to violent storm and hurricane force events by U.S. county, 1988–2019.

There were 179 named Atlantic basin tropical cyclones during 1988–2019 (Fig. 1), of which (i) 109 named tropical cyclones contributed (i.e., at least one tropical cyclone–force count in at least one county) to 5180 tropical cyclone county-months in 1258 counties and (ii) 36 named tropical cyclones (of 109) contributed (i.e., at least one hurricane-force count in at least one county) to 229 hurricane county-months in 154 counties (Table 1). Total tropical cyclone–force counts in a single county across all years ranged from 1 to 27 county-months, with a median of 2 (mean: 4.2) (Fig. 2). Total hurricane-force counts in a single county across all years ranged from 1 to 5 county-months, with a median of 1 (mean, 1.5). Tropical cyclone county-months occurred from May to November, with the greatest monthly occurrence in September (2173 county-months). Hurricane county-months occurred from July to October, with greatest occurrence in September (94 county-months). Tropical cyclones were most frequent in the eastern and southeastern coastal counties.

**Table 1.** Tropical cyclones from 1988–2019 and their maximal intensities recorded in the United States (in knots), with annual number of tropical cyclone, gale to violent storm, and hurricane county-months in the United States. Underlined tropical cyclone names were subsequently retired by the World Meteorological Organization because of destruction wreaked in the United States or elsewhere.

Year	Tropical cyclones [maximal intensity in the United States (knots)]	Tropical cyclone county-months	Gale to violent storm county-months	Hurricane county-months
1988	Alberto (22.8), Beryl (44.8), Chris (34.1), Florence (51.4), Gilbert (33.0), Keith (47.8), AL13 (11.3), AL14 (27.8), AL17 (12.6)	54	54	0
1989	Allison (45.0), Chantal (61.3), Hugo (94.0), Jerry (65)	246	235	11
1990	AL01 (16.7), Bertha (11.0), Marco (46.7)	12	12	0
1991	Ana (20.0), Bob (75.6), Fabian (22.8), AL12 (30.9)	102	94	8
1992	AL02 (25.0), Andrew (110.1), Danielle (46.8), Earl (19.3)	101	88	13
1993	AL01 (9.8), Arlene (30.6), Emily (57.9)	35	35	0
1994	Alberto (50.0), AL02 (27.2), Beryl (49.4), Gordon (50.5)	55	55	0
1995	Allison (52.2), Dean (34.4), Erin (72.5), Gabrielle (20.7), Jerry (35.0), Opal (82.9)	283	274	9
1996	Arthur (25.5), Bertha (81.8), Edouard (34.4), Fran (88.3), Josephine (49.5)	386	373	13
1997	AL01 (19.0), Ana (12.3), Danny (62.8)	62	62	0
1998	Bonnie (92.1), Charley (36.4), Earl (64.8), Frances (43.6), Georges (83.2), Hermine (30.6), Mitch (51.5)	300	284	16
1999	Bret (91.8), Dennis (54.5), AL07 (16), Floyd (75.8), Harvey (44.7), Irene (65.0)	225	210	15
2000	AL04 (17.8), Beryl (25.5), AL09 (24.4), Gordon (49.4), Helene (39.9), Leslie (29.9)	37	37	0
2001	Allison (41.3), Barry (55.1), Gabrielle (54.9), Karen (14.7), Michelle (24.6)	70	70	0
2002	Arthur (20.3), Bertha (31.0), Cristobal (13.4), Edouard (31.3), Fay (44.8), Gustav (36.9), Hanna (42.4), Isidore (50.5), Kyle (31.6), Lili (69.9)	89	86	3
2003	Bill (45.3), Claudette (65.9), AL07 (21.5), Erika (47.9), Grace (34.7), Henri (28.3), Isabel (81.7)	227	217	10
2004	Alex (51.7), Bonnie (29.9), Charley (107.3), Frances (79.9), Gaston (59.3), Hermine (32.3), Ivan (90.0), Jeanne (94.8), Matthew (26.2)	227	203	24
2005	Arlene (45.9), Cindy (47.9), Dennis (95.1), Emily (45.3), Katrina (94.2), Ophelia (54.6), Rita (82.0), Tammy (39.2), Twenty-Two (27.0), Wilma (79.6)	263	233	30
2006	Alberto (38.3), Beryl (34.3), Chris (9.3), Ernesto (47.0)	114	114	0
2007	Andrea (21.9), Barry (38.2), Erin (47.7), Gabrielle (39.8), Humberto (67.6), Ten (24.2), Noel (31.0)	90	88	2
2008	Cristobal (31.7), Dolly (64.3), Edouard (52.2), Fay (58.7), Gustav (87.1), Hanna (55.2), Ike (88.8), Kyle (35.3), Paloma (8.9)	544	523	21
2009	One (10.4), Claudette (38.2), Ida (30.7)	6	6	0
2010	Alex (31.8), Two (24.8), Bonnie (30.6), Five (24.9), Earl (31.4), Hermine (51.8), Nicole (20.0), Paula (16.1)	22	22	0
2011	Bret (18.4), Don (26.6), Emily (14.3), Irene (65.1), Lee (39.9)	193	191	2
2012	Alberto (25.2), Beryl (51.0), Debby (30.3), Isaac (63.3), Sandy (64.5)	196	195	1
2013	Andrea (40.1), Dorian (16.0), Karen (16.9)	80	80	0
2014	Arthur (74.4)	55	53	2
2015	Ana (38.7), Bill (49.9), Claudette (19.7)	19	19	0
2016	Bonnie (29.1), Colin (44.9), Eight (16.5), Hermine (63.1), Julia (44.1), Matthew (68.0)	272	271	1
2017	Cindy (44.1), Emily (37.4), Harvey (103.1), Irma (91.9), Jose (23.9), Nate (60.6), Philippe (8.6)	167	150	17
2018	Alberto (36.8), Chris (15.7) Florence (79.3), Gordon (57.4), Michael (130.6)	352	321	31
2019	Barry (61.3), Dorian (58.2), Fernand (18.6), Imelda (33.3), Melissa (20.4), Nestor (33.9), Olga (44.9), Three (18.1)	296	296	0
**Total**		**5180**	**4951**	**229**

**Fig. 3.**
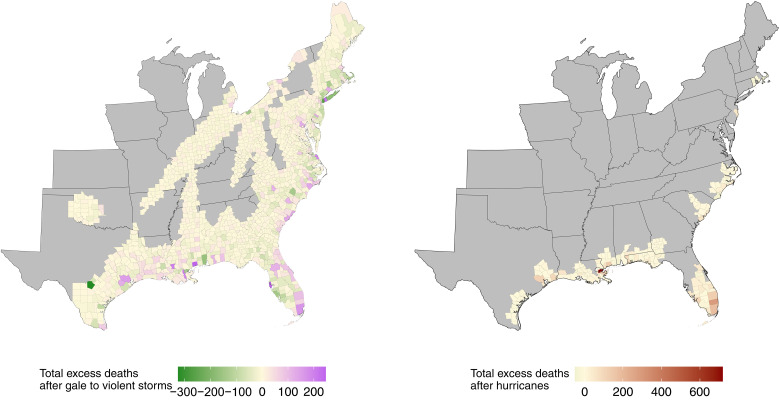
Total estimated excess deaths after tropical cyclones categorized by gale to violent storm force and hurricane force events by U.S. county, 1988–2019. Please note that the scale of each map is distinct.

There were 40,689,275 total deaths during 1988–2019 in the 1258 counties that experienced at least one tropical cyclone. There was substantial variation in the number of excess deaths by year and county in the month of and in the month after tropical cyclones, from either gale to violent storm or hurricane force winds (Fig. 3). The largest annual county-level excess death count was in Orleans Parish, LA in 2005, with 719 [95% credible interval (CrI): 685, 752] estimated total post–tropical cyclone excess deaths; followed by Harris County, TX in 2005, with 309 [95% CrI: 182, 429] estimated total post–gale to violent storm deaths; and Broward County, FL in 2016, with 185 [95% CrI: 86, 276] estimated total post–gale to violent storm deaths. In counties in years where the posterior probability that excess deaths were more than zero (referred to as “probability” hereafter) was >95% [73 hurricane force county-years (33.5% of total) and 952 gale to violent storm force county-years (21.4% of total)], there were a total of 3112 [95% CrI: 2451, 3699] estimated excess deaths after hurricane force winds and 15,590 [95% CrI: 12,084, 18,835] after gale to violent storm force winds. There were 42 county-years (19.3% of total) where the probability of excess deaths was <5% after hurricane force winds and 1232 county-years (27.7% of total) after gale to violent storm force winds. Table S1 lists the estimated excess deaths for the top 20 most deadly county-years by year, tropical cyclone category, and county.

The most estimated excess deaths in a single year were during 2005, with 2163 [95% CrI: −97, 4105] estimated post–tropical cyclone excess deaths (97.0% probability), consisting of 1637 [95% CrI: 996, 2220] estimated post–tropical cyclone excess deaths (>99% probability) and 516 [95% CrI: −1105, 1924] estimated post–gale to violent storm deaths (74.0% probability). In counties in years where the probability was >95%, 72.2% (13,101 [95% CrI: 9532, 16,382] estimated excess deaths) of the total estimated post–tropical cyclone excess deaths (18,158 [95% CrI: 14,267, 21,716] estimated excess deaths) occurred in the latter half of our study period (2004–2019). In counties in years where the probability was <5%, 616 county-years (49.1% of total) occurred in the latter half of our study period (2004–2019).

The largest annual estimated excess death count in a single state was in Florida during 2017 with 1403 [95% CrI: −259, 2956] estimated post–tropical cyclone excess deaths (95.3% probability), followed by Louisiana during 2005, with 1238 [95% CrI: 840, 1582] estimated post–tropical cyclone excess deaths (>99% probability). The largest annual post–hurricane force wind excess death count in a single state was in Louisiana in 2005 (1005 [95% CrI: 895, 1100] estimated excess deaths; >99% probability) followed by Florida in 2004 (404 [95% CrI: 45, 749] estimated excess deaths; 98.4% probability). The probabilities following gale to violent storms were often lower than 95%; the highest was in Texas in 2005 (502 [95% CrI: 225, 759] excess deaths; >99% probability), followed by Louisiana in 2017 (274 [95% CrI: 4, 516] excess deaths; 97.6% probability). The states with the largest number of years with a greater than 95% probability of post–tropical cyclone excess deaths were Florida (2002 and 2017), Georgia (2006 and 2008), Louisiana (2005 and 2017), and Texas (1995 and 2005), with Florida for post-hurricane excess deaths (1999, 2004, and 2017) and Georgia (2006 and 2008), Louisiana (2008 and 2017), and Texas (1995 and 2005) for post–gale to violent storm deaths.

**Fig. 4.**
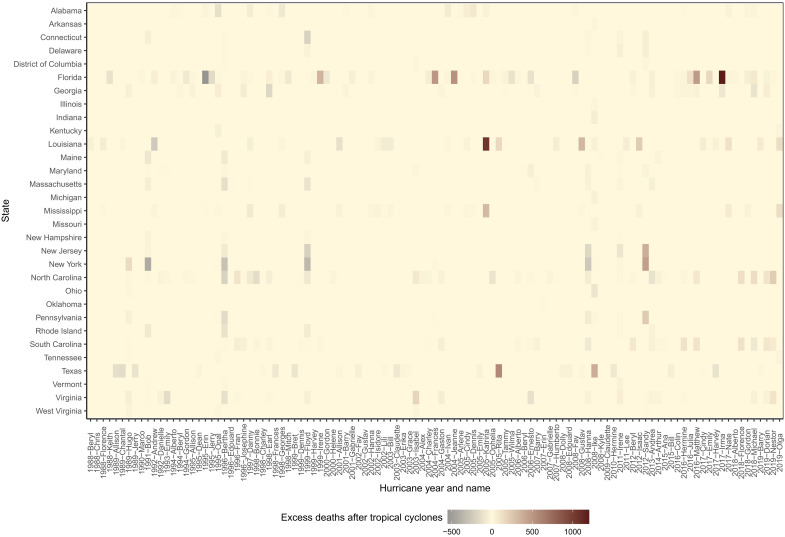
Total estimated excess deaths by hurricane and year, 1988–2019. Only states that had at least one tropical cyclone exposure during 1988–2019 are included.

The number of post–tropical cyclone excess deaths varied by tropical cyclone. The deadliest tropical cyclone was Hurricane Katrina in 2005, for which there were an estimated 1491 [95% CrI: 563, 3206] post–tropical cyclone excess deaths (>99% probability) (Fig. 4). The second deadliest was Hurricane Irma in 2017 with 1202 [95% CrI: −381, 2662] estimated post–tropical cyclone excess deaths (94.0% probability), with 147 [95% CrI: 2, 292] estimated excess deaths in Miami-Dade County, FL (98% probability); 134 [95% CrI: 29, 235] in Palm Beach County, FL (>99% probability); and 106 [95% CrI: 49, 157] in Lee County, FL (>99% probability). The third was Hurricane Sandy in 2012 with 1193 [95% CrI: −1996, 3826] estimated post–tropical cyclone excess deaths (78.7% probability), with 178 [95% CrI: 67, 271] estimated excess deaths in Nassau County, NY (>99% probability); 104 [95% CrI: 56, 143] in Richmond County, NY (>99% probability); and 54 [95% CrI: 11, 90] in York County, PA (>99% probability).

Table 2 lists the estimated excess deaths for the top 20 most deadly county–tropical cyclones. The largest estimated excess death count in a single county after a tropical cyclone was for Orleans Parish, LA after Hurricane Katrina in 2005 (719 [95% CrI: 685, 752] estimated excess deaths; >99% probability); followed by Harris County, TX after Hurricane Rita in 2005 (309 [95% CrI: 182, 429] estimated excess deaths; >99% probability); and Broward County, FL after Hurricane Matthew in 2016 (185 [95% CrI: 86, 276] estimated excess deaths; >99% probability).

**Table 2.** Estimated hurricane-specific county excess deaths after the top 20 most deadly tropical cyclone events, with wind category and precipitation (mm/day).

Rank	Year	Hurricane name	County	Estimated excess deaths	Wind category	Maximal precipitation (mm/day)	Posterior probability*
1	2005	Katrina	Orleans Parish, LA	719 (685,752)	Hurricane	191	>99%
2	2005	Rita	Harris County, TX	309 (182,429)	Gale to violent storm	23	>99%
3	2016	Matthew	Broward County, FL	185 (86,276)	Gale to violent storm	3	>99%
4	2012	Sandy	Nassau County, NY	178 (67,271)	Gale to violent storm	2	>99%
5	1999	Irene	Broward County, FL	167 (70,258)	Hurricane	110	>99%
6	2017	Irma	Miami-Dade County, FL	147 (2,292)	Gale to violent storm	136	97.6%
7	2005	Katrina	Harrison County, MS	141 (126,156)	Hurricane	129	>99%
8	2005	Katrina	St. Bernard, LA	138 (128,148)	Hurricane	179	>99%
9	1992	Andrew	Miami-Dade County, FL	136 (9,249)	Hurricane	75	98.3%
10	2017	Irma	Palm Beach County, FL	134 (29,235)	Gale to violent storm	109	98.9%
11	2016	Matthew	Miami-Dade County, FL	119 (−20,256)	Gale to violent storm	3	95.5%
12	2008	Ike	Harris County, TX	117 (−22,255)	Hurricane	129	94.8%
13	2017	Irma	Lee County, FL	106 (49,157)	Hurricane	63	>99%
14	2005	Wilma	Broward County, FL	104 (5,191)	Hurricane	40	98.1%
15	2012	Sandy	Richmond County, NY	104 (56,143)	Gale to violent storm	2	>99%
16	1999	Irene	Miami-Dade County, FL	95 (−32,221)	Gale to violent storm	156	93.0%
17	2005	Katrina	Miami-Dade County, FL	93 (−29,209)	Hurricane	61	93.6%
18	2017	Irma	Pinellas County, FL	84 (−18,179)	Gale to violent storm	107	95.5%
19	2012	Sandy	King’s County, NY	84 (−75,220)	Gale to violent storm	1	85.1%
20	2017	Irma	Broward County, FL	81 (−20,173)	Gale to violent storm	121	94.0%

*Excess deaths were >0.

**Fig. 5.**
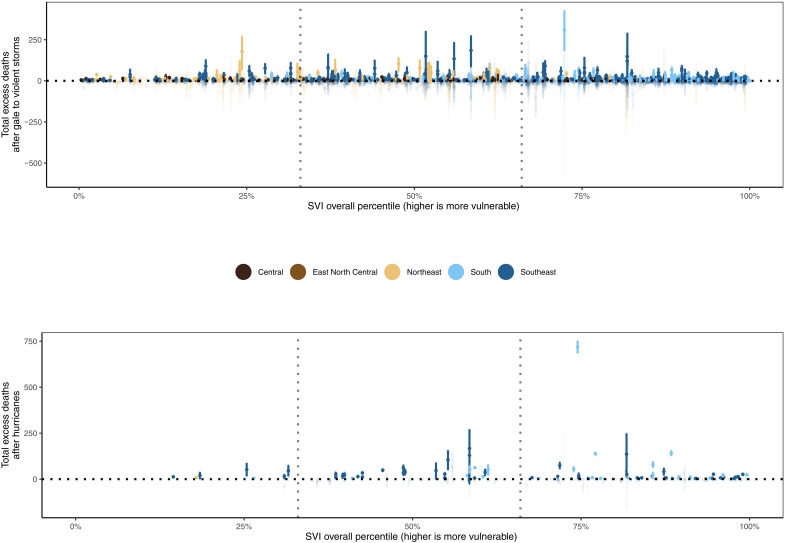
Estimated annual county-level excess deaths after tropical cyclones categorized by gale to violent storm force and hurricane force events against SVI percentile, 1988–2019. Dots show the point estimates, and whiskers represent 95% credible intervals. Vertical dotted lines represent boundaries of SVI tertiles. Highlighted points represent counties with a posterior probability of >95% of excess deaths.

The post–tropical cyclone excess death count also varied by social vulnerability (Fig. 5). Of the 1258 counties experiencing at least one tropical cyclone force wind during our study period, 277 counties were in the least vulnerable Social Vulnerability Index tertile 1 (SVI-t1), 408 were in SVI-t2, and 573 were in the most vulnerable SVI-t3. There were also regional variations in the composition of SVI tertiles; counties included in our analysis that were in SVI-t3 were largely from states in the southeast (275 counties; 48% of counties in SVI-t3) and south (184 counties; 32.1% of counties in SVI-t3), compared with SVI-t2 with states in the southeast (133 counties; 32.6% of counties in SVI-t2) and central (127 counties; 31.1% of counties in SVI-t2) and SVI-t1 with states in the northeast (96 counties; 34.7% of counties in SVI-t1) and central (86 counties; 31% of counties in SVI-t1).

For hurricane force events, in counties where the probability was >95%, the greatest total excess death count was in the highest vulnerability SVI tertile (SVI-t3) (1781 [95% CrI: 1529, 2016] estimated excess deaths; 57.2% of excess deaths; >99% probability that SVI-t3 excess death count was greater than SVI-t1), followed by SVI-t2 (1139 [95% CrI: 761, 1480] estimated excess deaths; 36.6% of excess deaths; >99% probability that SVI-t2 excess death count was greater than SVI-t1) and SVI-t1 (193 [95% CrI: 97, 290] estimated excess deaths; 6.2% of excess deaths). There was an 85.4% probability that SVI-t3 excess death count was greater than SVI-t2.

For gale to violent storm force events, in counties where the probability was >95%, the greatest total excess death count was in the highest vulnerability SVI-t3 (7726 [95% CrI: 5882, 9384] estimated excess deaths; 49.6% of excess deaths; >99% probability that SVI-t3 excess death count was greater than SVI-t1), followed by SVI-t2 (5192 [95% CrI: 3983, 6308] estimated excess deaths; 33.3% of excess deaths; >99% probability that SVI-t2 excess death count was greater than SVI-t1) and SVI-t1 (2654 [95% CrI: 2033, 3254] estimated excess deaths; 17.0% of excess deaths). There was an 89.6% probability that SVI-t3 excess death count was greater than SVI-t2. Excess deaths in SVI-t2 and SVI-t3 were consistently higher than in SVI-t1 when considering SVI percentiles within each state for the top four most affected states (Florida, Georgia, Louisiana, and North Carolina), all located in the southeast (fig. S1 and table S2). When considering individual SVI components, the proportion of hurricane force excess deaths for racial and ethnic minority status SVI tertiles was relatively higher in SVI-t3 (87.6%) than SVI-t1 (2.5%) when compared to the overall SVI (and similar for gale to violent storm force), but relatively lower for household characteristics SVI tertiles in SVI-t3 (24.0%) than SVI-t1 (56.3%) (and similar for gale to violent storm force) (figs. S2 to S6 and table S3).

## DISCUSSION

Among U.S. counties that experienced at least one tropical cyclone during 1988–2019, there was a large variation in cyclone-related excess deaths by state, county, year, and social vulnerability. The same tropical cyclone can affect communities differently, with impacts likely driven by demographic, economic, and social factors (*12*), often from systemic inequity (*13*). Residents in low-income and historically disadvantaged communities have been differentially affected after disasters (*14*). In our study, we also estimated fewer excess deaths after tropical cyclones in the least socially vulnerable counties compared with the more socially vulnerable counties, although counties with low vulnerability are also in the path of tropical cyclones less often. Disparities in post-disaster mortality remain pervasive, with higher death rates in groups such as older adults and males of American Indian/Alaska Native and Black origin relative to other groups (*15*). This is in part due to not only lack of access to adequate short-term transportation but also inequitable access to financial resources, education, employment opportunities, and timely warnings on tropical cyclone proximity (*15*). Differences in the county-level impact of a tropical cyclone are also driven by variations in the demographic structure of each county, including the age distribution of residents, as well as prevalence of residents living with preexisting chronic health issues, such as neuropsychiatric conditions (*16*). The finding of more excess deaths in the most vulnerable racial and ethnic minority status tertiles further suggests that institutional historical neglect plays a role in the number of excess deaths in the immediate aftermath of a tropical cyclone. Many of the most socially vulnerable counties in the United States are in the south and southeast, areas that are also most exposed to tropical cyclones, compounding risk of death through greater relative exposure and vulnerability.

We also compared the estimated excess deaths in our study with data from official sources and the Emergency Event Database (EM-DAT), recognizing that they may not be directly comparable as excess deaths accounts for deaths in those who would have lived at least for the month of and for the month after a cyclone; official figures may include all regardless of whether they were about to die (*17*). Our results agree well with estimates of attributable deaths after Hurricane Katrina, the deadliest tropical cyclone estimated in our study, although our estimates are higher; we estimated 1491 post–tropical cyclone excess deaths, and the official figure is 1170 (*18*). In some cases, our estimates of excess deaths are considerably higher; for Hurricane Irma in 2017, there were an estimated 1206 post–tropical cyclone excess deaths, while the official figure is 97 (*19*). In some limited cases, our estimates were slightly lower; for Hurricane Andrew in 1992, there were an estimated 40 post–tropical cyclone deaths in Florida, while the official figure is 44 (*19*), which may indicate that some of those who died may have been already close to death. There are many reasons that excess deaths may vary from official counts, including different methods of attributing direct and indirect deaths via death certificates, as well as other potentially bureaucratically and politically motivated reasons to minimize counts.

Leveraging complete death data from 40.7 million deaths and a tropical cyclone dataset over a 32-year period, our study is a comprehensive investigation of excess mortality after tropical cyclones, a key recommended measure for post-disaster mortality assessment (*6*, *7*), using a consistent method over space and time. This is in addition to and is distinct from our previous work that detailed the causes of death for which the risk increased after tropical cyclones (*5*). Nevertheless, this study has several limitations. First, a potential limitation is exposure misclassification (*20*). Exposure misclassification, however, is likely nondifferential as it is not expected to be correlated with the outcomes assessed (*20*). Furthermore, the tropical cyclone exposure data are well validated, with no evidence of exposure estimation varying in space (*21*). Second, this study focused on the continental United States (CONUS), although devastating effects of tropical cyclones, such as Hurricane Maria in Puerto Rico, have also been recorded (*10*). This was in part because currently publicly available, curated tropical cyclone, temperature, and precipitation data only cover the CONUS, with SVI also covering CONUS and Puerto Rico; further work should increase the scope to other U.S. territories and other parts of the world. Third, the unit of analysis was U.S. county, but counties contain disparities within them, which was not captured in our analysis. Future work may be able to study associations by smaller areal units, e.g., ZIP codes, as appropriate exposure and outcome data become available. Fourth, analysis of social vulnerability used 2018 values for the entire study period; however, rankings of social vulnerability change over time, although there was a high correlation of social vulnerability when comparing the first available (2000) and the last available (2018) years (*5*). SVI is also an imperfect measure of social vulnerability, and results should be understood in that context (*22*–*25*). Further work should examine the impact of repeated tropical cyclone exposure on social vulnerability over time. Fifth, the data used extended only through 2019, and it is unknown whether the findings of this study reflect more recent cyclone activity and mortality following cyclones. Sixth, although tropical cyclones are particularly devastating events in comparison to other climate-related exposures and we robustly tested our model validity, we cannot completely rule out whether there were other unrelated deadly co-occurring hazards, partly expressed by the uncertainty in our excess death estimates. Seventh, our analysis examined deaths in the month of and in the month following a tropical cyclone, but tropical cyclone exposure may result in deaths many years or decades later, outside the scope of our study design.

Our work highlights how deaths are affected by tropical cyclones, an understudied exposure in relation to public health and one which will remain an important threat as the climate continues to change (*26*). It is essential to prepare for tropical cyclones by accounting for the social determinants of risk and vulnerability of exposed communities, because the most socially vulnerable bears the greatest burden of excess mortality. Trust and awareness of tropical cyclone warnings and the capacity to act upon warnings, for example, by temporarily evacuating an area, are other important factors driving the variation in numbers of excess deaths in the immediate aftermath (*27*). Non–hurricane force tropical cyclones occur more frequently than hurricane force ones and were associated with more total excess deaths, which may be important for local and state authorities to consider beyond hurricane force winds alone. As a public health priority, future research should focus on understanding the biological and structural drivers of cyclone-related mortality, how to minimize the number of excess deaths related to tropical cyclones, and the impacts on the scale from years to decades.

## MATERIALS AND METHODS

### Study design

Our aim was to estimate the number of excess deaths after tropical cyclones in the United States by county, year, named hurricane, and strength. Knowledge of the total excess deaths is essential for understanding the true public health burden of powerful tropical cyclones, such as named hurricanes (*28*). However, methodology to do this has been hitherto inconsistent (*29*); even estimates of the same hurricane can vary greatly, such as Hurricane Maria in 2017, for which official death counts were up to 70 times lower than the total number of excess deaths (*10*, *30*).

To address this critical research gap, we estimated the number of excess deaths directly from complete mortality records. We formulated a Bayesian ensemble of 16 forecasting models each with different model parameterizations and a model-averaging approach to estimate counterfactual county-level monthly death counts had a tropical cyclone not occurred in the month of and in month after a tropical cyclone. We compared actual death rates to counterfactual rates and used population estimates to calculate the number of excess deaths. We categorized excess deaths after gale to violent storm and hurricane force winds. An example of this approach for Orleans County, LA is found in fig. S7.

### Exposure assessment

We obtained data on tropical cyclone wind exposure in the United States, with full space and time coverage over our study period, described in detail elsewhere (*4*, *5*, *11*, *21*, *31*–*35*). Briefly, we used daily estimates of maximal wind sustained speed by county to generate classifications of these exposures. As in previous work (*4*, *5*), we defined tropical cyclone exposure as all days when the peak sustained wind that day in the population center of the county associated with the tropical cyclone at the point of closest approach reached or exceeded 34 knots (63 km/hour, 39 mph; gale force wind on the Beaufort scale), with gale to violent storm exposure as all days with greater than or equal to 34 knots but less than 64 knots and hurricane exposure all days with greater than or equal to 64 knots [119 km/hour, 74 mph; hurricane force wind on the Beaufort scale (*36*)]. A plot of best-track routes [subjectively smoothed representations of tropical cyclones’ location and intensity over their lifetimes (*37*)] of each tropical cyclone by year is found in Fig. 1. A full list of included tropical cyclones is found in Table 1, with a map of total tropical cyclone exposure counts in Fig. 2.

### Outcome assessment

We used data on all deaths by county of residence in counties that experienced at least one tropical cyclone exposure during 1988–2019 through the National Center for Health Statistics (NCHS) (*n* = 40,689,275; 100% of total deaths in tropical cyclone–exposed counties) (www.cdc.gov/nchs/nvss/dvs_data_release.htm) and on population from the NCHS Vintage 2020 bridged race dataset (although no analysis by race was carried out) for 1990 to 2019 (www.cdc.gov/nchs/nvss/bridged_race.htm) and from the U.S. Census Bureau before 1990 (www.census.gov/data/tables/time-series/demo/popest/1980s-county.html).

As in previous work (*5*, *38*, *39*), we calculated monthly population counts through linear interpolation, assigning each yearly count to June. We calculated death rates from death counts divided by the estimated population in each county-month. This study was approved by the Institutional Review Board at the Columbia University Mailman School of Public Health and was classified as exempt from needing to obtain informed consent (protocol IRB-AAAT9710).

### Covariate data

We obtained data on temperature from the Parameter-elevation Regressions on Independent Slopes Model (PRISM), which gathers climate observations from a wide range of monitoring networks and applies sophisticated quality control measures to generate a nationwide temperature dataset, with full space and time coverage over our study period (*40*). We used gridded daily estimates at a resolution of 4 km to generate area-weighted monthly average temperatures by county. We also used PRISM precipitation gridded daily estimates at a resolution of 4 km to generate daily average precipitation by county for use in the secondary and sensitivity analyses.

We used the latest available data on social vulnerability from the Centers for Disease Control and Prevention (CDC) SVI for 2018 (www.atsdr.cdc.gov/placeandhealth/svi/documentation/SVI_documentation_2018.html). The SVI incorporates data from the U.S. Census on four components to determine the relative social vulnerability of every U.S. county (*41*): (i) socioeconomic status (poverty, unemployed, housing cost burden, no high school diploma, and no health insurance), (ii) household characteristics (age, disability, single-parent households, and English language proficiency), (iii) racial and ethnic minority status (Hispanic or Latino, Black and African American, and American Indian), and (iv) housing type and transportation (multiunit structures, mobile homes, crowding, no vehicle, and group quarters). A county’s SVI value indicates the relative vulnerability of each county compared with every other county in the United States, ranking from 0% (county with the lowest vulnerability in the country) to 100% (county with the highest vulnerability in the country). Tools such as the CDC SVI are not perfect representations of social vulnerability (*22*) and are sensitive to the modeling choices for how they are constructed (*23*). CDC SVI has been well validated as a predictor of fatalities, while poorly validated for disaster declarations (*24*). On the basis of another study of Hurricane Sandy, CDC SVI was a poor predictor of housing damage and property loss (*25*).

We divided counties included in our analysis into SVI tertiles (low vulnerability to high vulnerability, 1 to 3; fig. S8). When comparing the first year of available SVI data (2000) (www.atsdr.cdc.gov/placeandhealth/svi/documentation/SVI_documentation_2000.html) to the SVI data we used from 2018 in our main analysis, we found a correlation of 0.89, and when only including the tropical cyclone–exposed counties in our study, we found a correlation of 0.90. When comparing mean SVI across 2000–2018 with 2018, we found a correlation of 0.98.

We subdivided the United States geographically into nine climate regions used by the National Oceanic and Atmospheric Administration (fig. S9) (*42*). For comparison, we obtained data on deaths associated with tropical cyclones from official state sources and the Emergency Event Database (EM-DAT).

### Statistical methods

We estimated the total mortality impact of a tropical cyclone (excess mortality) as the difference between the observed number of deaths from all causes and the counterfactual number of deaths had the tropical cyclone not passed through the United States. The counterfactual number of deaths, i.e., the number of deaths had the tropical cyclone not passed through, however, is not directly measurable.

To estimate the number of counterfactual deaths, we formulated a Bayesian ensemble of 16 predictive models, trained on periods of time within counties with no tropical cyclone exposure, to estimate monthly all-cause deaths for the month of and month after exposure in tropical cyclone-affected counties, defined as counties with a sustained maximal wind speed of ≥34 knots, had the cyclone not occurred. The models were designed by expanding those used to estimate excess mortality in recent previous work (*43*, *44*) and incorporated features of monthly death rates such as medium- to long-term trends, seasonal patterns (*38*), relation to immediately preceding months, and temperature anomalies (*39*). We compared actual deaths to estimated counterfactual deaths to calculate the number of excess deaths. We used multiple models because there is inherent uncertainty in the choice of model that best predicts death rates in the absence of a tropical cyclone, and model averaging improves overall prediction accuracy (*45*, *46*). We categorized excess deaths after gale to violent storm and hurricane force winds.

In each model, we assumed a Poisson distribution for the number of monthly deaths, also accounting for possible overdispersion. Using a log-link function, we modeled death counts for each county separately, as detailed in the equation below

$$\log(E[{\mathrm{deaths}}_{\mathrm{time}}])=\alpha_{0}+\theta_{\mathrm{month}}+\zeta_{\mathrm{month}}^{\left(i\right)}+(\beta +\omega_{\mathrm{month}})*\mathrm{time}+(\gamma +\nu_{\mathrm{month}})*{\text{temperature anomaly}}_{\mathrm{month}}+\log({\mathrm{Population}}_{\mathrm{time}})+\varepsilon_{\mathrm{time}}$$

These models were formulated to incorporate features of monthly death rates as follows: First, there is the initial value, or overall intercept, of death rates at the beginning of our study period (January 1988). The term α_0_ denotes the overall intercept fixed effect, to which we assigned *N*(0,1000) prior.

Second, there are long-term trends in death rates, in part, driven by long-term air pollution concentrations (*47*). We developed two sets of models, one assuming no trend and one with a linear trend term over monthly deaths, as in previous similar work for annual and weekly death rates (*43*, *44*, *48*, *49*). The term β*time represents the linear time trend fixed effect. The coefficient β was also assigned *N*(0,1000) prior. This term appeared in half of our models, whereas in the other half, trends over time were captured by the remaining terms (i.e., β = 0).

Third, death rates in the United States have a seasonal pattern (*38*). We included monthly random intercepts for each month of the year. To account for the fact that seasonal patterns do not reset at the end of each year (that is, late December and early January are seasonally similar), we used a seasonal structure for the random intercepts (*50*, *51*). The seasonal structure allows the magnitude of the random intercepts to vary over time and implicitly incorporates time-varying factors, such as annual fluctuations in flu season. The random seasonal intercept θ_month_ captures seasonality in mortality trends with a period of 12 months. The sums of every 12 consecutive terms θ_month_ + θ_month+1_ + … + θ_month+11_ were modeled as independent Gaussian with zero mean and variance $\sigma_{\theta }^{2}$. We used a logGamma(0.001,0.001) prior on the log precision $\log(1/\sigma_{\theta }^{2})$. Each month was assigned an index between 1 and 12. The random seasonal slope term ω_month_ captures the long-term change in seasonality in mortality trends (i.e., change over time from the initial seasonal pattern in first study year, 1988) with a period of 12 months, specified in the same way as θ_month_ but as a slope over time.

Fourth, death rates in each month might be related to rates in preceding month(s) because of short-term phenomena (*17*). We formulated four sets of models to account for this relationship. The monthly random intercepts in these models had a first-, second-, fourth-, or sixth-order autoregressive structure (*50*, *51*). The higher-order autoregressive models allow death rates in any given month to be informed by those in a progressively larger number of preceding months. Furthermore, trends not picked up by the linear or seasonal terms would be captured by these autoregressive terms. The models used different orders (first, second, fourth, or sixth) of the autoregressive term $\zeta_{\mathrm{month}}^{\left(i\right)}$, with the superscript *i* denoting the order. The first-order autoregressive term was defined as $\zeta_{\mathrm{month}}^{\left(1\right)}∼N(\varphi^{*}\zeta_{\mathrm{month}-1}^{\left(1\right)},\sigma_{\zeta }^{2})$, where the parameter ϕ lies between −1 and 1 and captures the degree of association between the number of deaths in each month and the preceding month. For this specification of the autoregressive structure (see https://inla.r-inla-download.org/r-inla.org/doc/latent/ar1.pdf), hyperpriors were placed on the parameters $\kappa_{1}=\log[(1-\varphi^{2})/\sigma_{\zeta }^{2}]$ and ϰ_2_ = log [(1 + ϕ)/(1 − ϕ)], which were assigned logGamma(0.001,0.001) and *N*(0,1) distributions, respectively. Similarly, an *i*th-order autoregressive term is given by $\zeta_{\mathrm{month}}^{\left(1\right)}=\varphi *\zeta_{\mathrm{month}-1}^{\left(i\right)}=\varphi_{1}*\zeta_{\mathrm{month}-1}^{\left(i\right)}+\cdots +\varphi_{i}*\zeta_{\mathrm{month}-i}^{\left(i\right)}+\varepsilon_{\mathrm{month}}$ with −1 < ϕ*_j_* < 1.

Fifth, beyond having a seasonal pattern, temperature is an important environmental predictor of death rates (*52*, *53*) and, specifically, whether temperature is higher or lower than its long-term norm during a particular time of year (*39*, *54*–*57*). Other time-varying factors influencing death rates throughout the year, such as flu season, would be captured by nonlinear autoregressive terms. The effect of temperature on mortality varies throughout the year. We used two sets of models, one without temperature and one with terms to account for temperature anomaly, defined as deviation of monthly temperature from the local average monthly temperature over the entire analysis period. The effect of temperature anomaly on death rates was captured by the two terms γ (fixed effect) and ν_month_ (random effect). The term γ∗temperature anomaly_month_ is the overall association of temperature anomaly in a month. The term ν_month_ ∗ temperature anomaly_month_ captures deviations from the overall association for each month of the year. The fixed term γ was assigned *N*(0,1000) prior. The coefficients of month-specific temperature anomalies were specified as a random effect with a random walk prior of order one, so that temperature effects are more similar in adjacent months. The month-specific random effect had a circular first-order random walk with 12 terms so that temperature associations changed smoothly throughout the year and so that they were similar in, e.g., December and January (*39*). The first-order random walk prior was defined via ν_month_~ $N(\nu_{\mathrm{month}-1},\sigma_{\nu }^{2})$, and the prior assigned to the log precision was $\log(1/\sigma_{\theta }^{2})∼\log\mathrm{Gamma}(0.001,0.001)$.

Sixth, the term log(Population_time_) is the model offset, using the interpolated monthly population in each county. Last, the term ɛ_time_ is a zero-mean term that accounts for overdispersion. It was assigned an independent and identically distributed prior ɛ_time_, and a logGamma(0.001, 0.001) prior was placed on the $\log(1/\sigma_{\varepsilon }^{2})$.

These choices led to an ensemble of 16 Bayesian models (2 trend options × 4 autoregressive options × 2 temperature options). The ensemble of models is shown in table S4. The components α_0_, θ_month_, ɛ_time_, and $\zeta_{\mathrm{month}}^{\left(i\right)}$ (for each autoregressive order of *i* = 1, 2, 4, or 6) appear in the expression for log(*E*[deaths_time_]) in all models. The remaining components appear in some models only. Table S4 shows the terms included in each of the 16 models in the ensemble.

We used data on monthly deaths from the start of the time series of data (January 1988) to the end of the time series data (December 2019) to estimate the parameters of each model, i.e., we trained each model to infer the parameters from periods of time within counties with no tropical cyclone exposure. We then used these trained models to predict death rates for the month of and month after tropical cyclone exposure in a county as estimates of the counterfactual death rates (that is, had the tropical cyclone not occurred). For the counterfactual periods, we used recorded temperature so that our counterfactual estimates took into consideration actual recorded temperatures. This choice of training and prediction periods assumed that the number of deaths that were directly or indirectly related to the tropical cyclone was largely negligible past a month after the tropical cyclone passed through these counties, based on previous work (*5*).

All models were fitted using integrated nested Laplace approximation (INLA) (*58*), implemented in the R-INLA software (version 20.03.1). We used a model-averaging approach to combine the predictions from the 16 models in the ensemble (*45*, *46*). Specifically, we took up to 1000 draws from the posterior distribution of deaths from each ensemble model to obtain the posterior distribution of deaths if the tropical cyclone had not occurred. To weight each model contributing to the ensemble by its predictive accuracy, the number of draws we took from each model was related to the mean percentage error of the model in the validation period, described in the “Validation” section below. Specifically, we took draws from the posterior of the estimated counterfactual death rates from each model during a tropical cyclone proportional to the inverse of the percentage error, with a maximum of 1000 and a minimum of 0 draws, as reported in table S4. The reported credible intervals represent the 2.5th and 97.5th percentiles of the resulting posterior distribution of the draws from the entire ensemble. We also report the posterior probability that an estimated increase in deaths corresponds to a true increase. A map of the posterior probability that excess deaths were >0 after tropical cyclones during our study period can be found in fig. S10.

A list of estimated excess deaths for the top 20 most deadly county–tropical cyclones is available in Table 2. A list of estimated excess deaths for the top 20 most deadly county-years by year, tropical cyclone category, and county is available in table S1.

### Validation

We tested how well the ensemble model estimated the number of deaths by withholding 2 months at a time (March and April of 1988 and March and April of 2019, respectively; periods without any tropical cyclone counts anywhere in the United States) of data and using the rest of the time series (January 1988 to December 2019 without March and April 1988 or March and April 2019) to train the models. We then used the ensemble output to predict death rates for the months of withheld data; we assessed the ensemble predictive error for each single ensemble member and the overall ensemble by comparing the predicted to observed death counts in the withheld data. We report the mean percentage error averaged over the two time periods from these validation runs in table S4. The mean percentage error for each of the 16 models was small, ranging from −0.37% (model 6) to 0.30% (model 10). The ensemble model yielded the lowest percentage error (−0.02%), compared to any single model (next smallest was 0.0205% for model 11).

### Secondary and sensitivity analyses

We tested the sensitivity of the estimated excess death to the choice of the validation period by using draws proportional to the inverse of the percentage errors either from validation period March and April of 1988 only or from validation period March and April of 2019 alone, instead of throughout the study period (main model). The mean difference between the main model and when using draws proportional to the inverse of the percentage errors from validation period March and April of 1988 was 0.03 deaths. The mean difference between the main model and when using draws proportional to the inverse of the percentage errors from validation period March and April of 2019 was 0.12 deaths (fig. S11).

We also estimated how consistent our model fit was for the counterfactual forecast using the entire time period (1988–2019; main model) compared with dividing the study period into separate counterfactual forecasts of the first half (1988–2003) and the second half (2004–2019). We then compared the agreement of the fits of each half compared with the entire period’s fit. The mean difference between the entire period’s excess death estimates and the first half’s excess death estimates was 0.70 deaths, and the mean difference between the entire period’s excess deaths and the second half’s excess deaths was 0.77 deaths (fig. S12).

In an effort to evaluate whether the observed differences by social vulnerability are fully attributed to regional differences, we repeated the SVI analyses in the four states most exposed to tropical cyclones throughout our study period (Florida, Georgia, Louisiana, and North Carolina), all in the southern United States, using SVI percentiles for each county relative to the state it belongs (fig. S1). Results were similar to Fig. 5 in that the least socially vulnerable counties within each of the four states bore the least mortality burden (table S2).

We also carried out the main SVI analysis (Fig. 4) for each of the individual SVI components (socioeconomic status, household characteristics, racial and ethnic minority status, and housing type and transportation) (figs. S2 to S5 and table S3). When considering individual SVI components, compared to the overall SVI analysis, the proportion of hurricane force excess deaths for racial and ethnic minority status SVI tertiles was relatively higher in SVI-t3 (87.6%) than SVI-t1 (2.5%) (and similar for gale to violent storm force), but relatively lower for household characteristics SVI tertiles in SVI-t3 (24.0%) than SVI-t1 (56.3%) (and similar for gale to violent storm force). A correlation plot of the overall SVI and SVI components for 2018 can be found in fig. S6.

We also plotted county-level daily precipitation (mm/day) against excess deaths by wind category to explore any potential association (fig. S13). We found no clear relationship between precipitation associated with tropical cyclones and excess deaths.
