# Supplementary material for: Short-term excess mortality following tropical cyclones in the United States
Source: Sci Adv. 2023 Aug 16;9(33):eadg6633. doi: 10.1126/sciadv.adg6633 (PMC10431701; doi:10.1126/sciadv.adg6633)
Supplement: Supplementary file 1 — Figs. S1 to S13 Tables S1 to S4 [file sciadv.adg6633_sm.pdf]

Science Advances
AAAS

Supplementary Materials for

Short-term excess mortality following tropical cyclones in the United States

Robbie M. Parks *et al.*

Corresponding author: Robbie M. Parks, robbie.parks@columbia.edu

Sci. Adv. **9**, eadg6633 (2023)
DOI: 10.1126/sciadv.adg6633

This PDF file includes:

Figs. S1 to S13
Tables S1 to S4

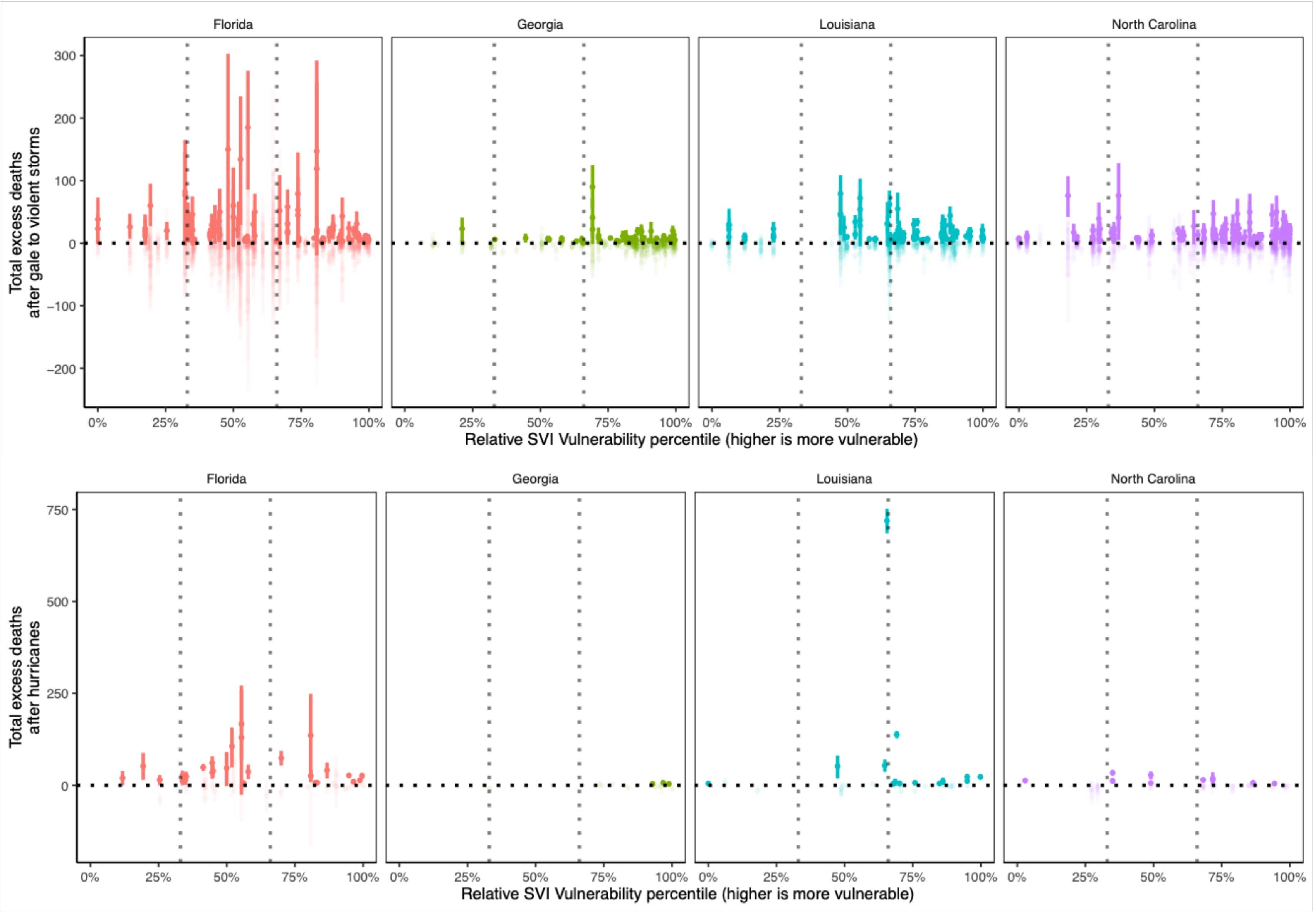

Fig. S1. Estimated annual county-level excess deaths after tropical cyclones categorized by gale to violent-force (top) and hurricane-force (bottom) events against Social Vulnerability Index (SVI) percentile, relative to rest of state, for top four most exposed states, 1988–2019. Vertical dotted lines represent boundaries of SVI tertiles. Highlighted points represent counties with a posterior probability>95% of excess deaths.

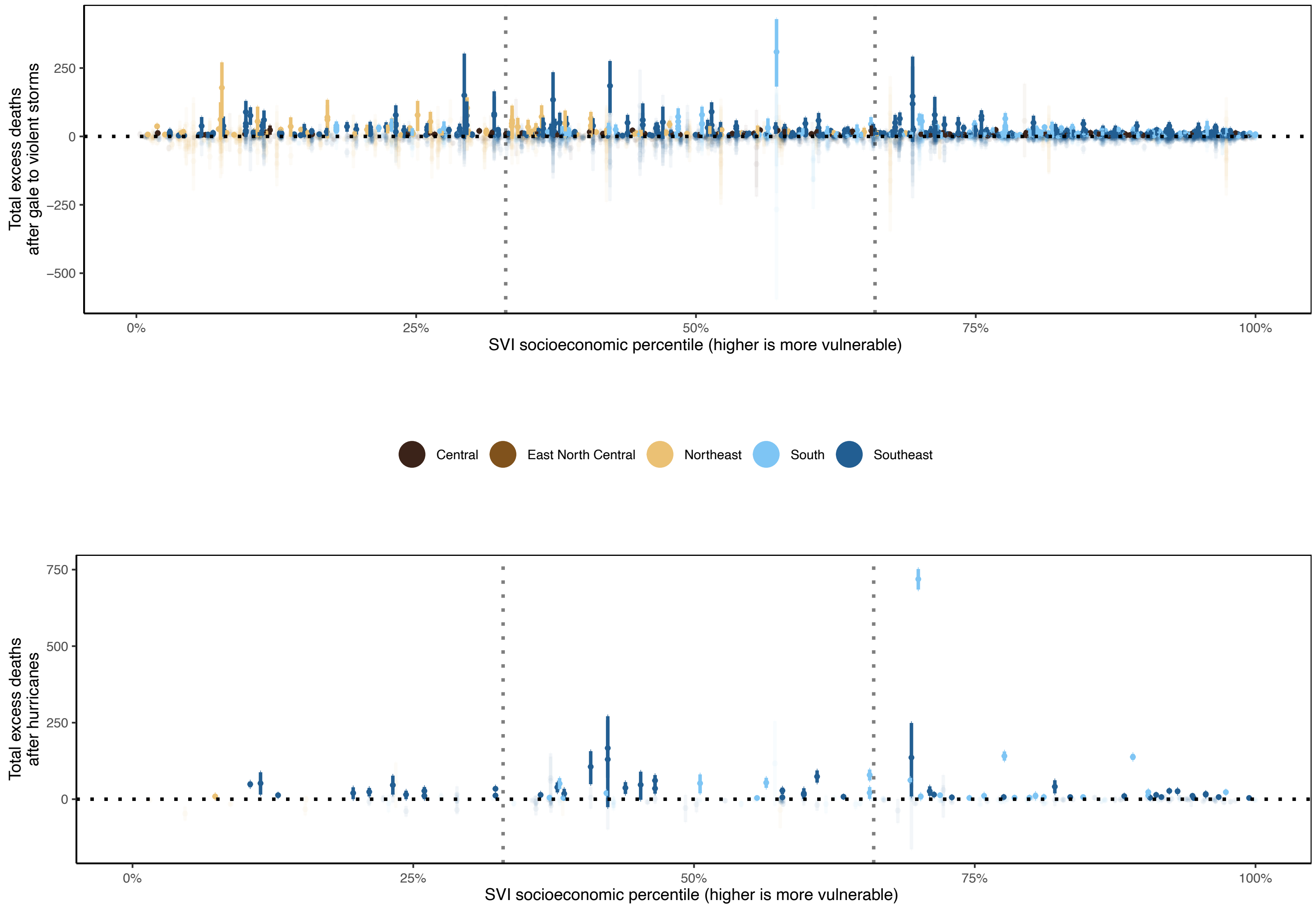

Fig. S2. **Estimated annual county-level excess deaths after tropical cyclones categorized by gale to violent-force (top) and hurricane-force (bottom) events against socioeconomic Social Vulnerability Index (SVI) percentile, 1988–2019.** Dots show the point estimates and whiskers represent 95% credible intervals. Vertical dotted lines represent boundaries of SVI tertiles. Highlighted points represent counties with a posterior probability>95% of excess deaths.

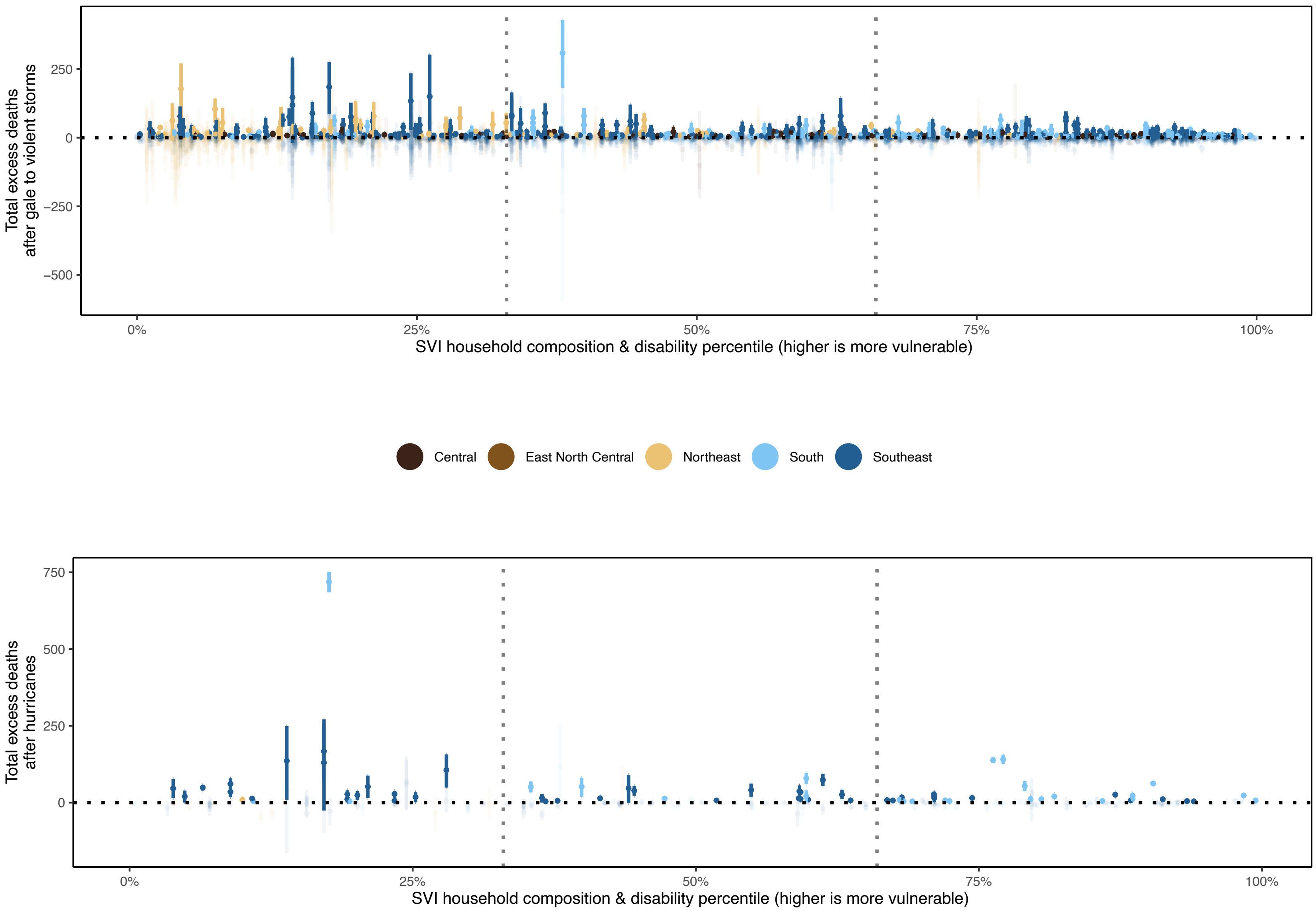

Fig. S3. Estimated annual county-level excess deaths after tropical cyclones categorized by gale to violent-force (top) and hurricane-force (bottom) events against household composition and disability Social Vulnerability Index (SVI) percentile, 1988–2019. Dots show the point estimates and whiskers represent 95% credible intervals. Vertical dotted lines represent boundaries of SVI tertiles. Highlighted points represent counties with a posterior probability>95% of excess deaths.

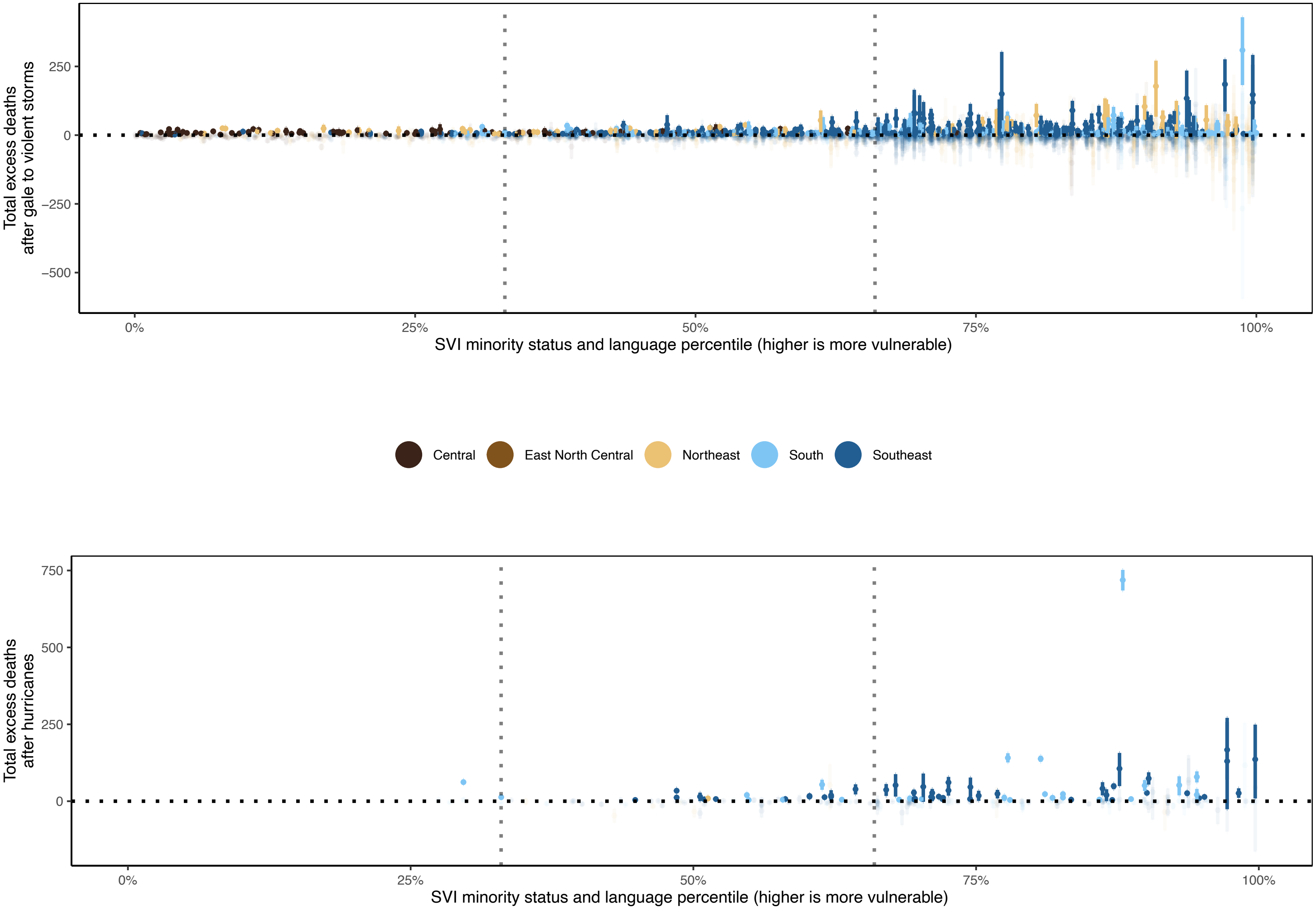

Fig. S4. **Estimated annual county-level excess deaths after tropical cyclones categorized by gale to violent-force (top) and hurricane-force (bottom) events against minority status and language Social Vulnerability Index (SVI) percentile, 1988–2019.** Dots show the point estimates and whiskers represent 95% credible intervals. Vertical dotted lines represent boundaries of SVI tertiles. Highlighted points represent counties with a posterior probability>95% of excess deaths.

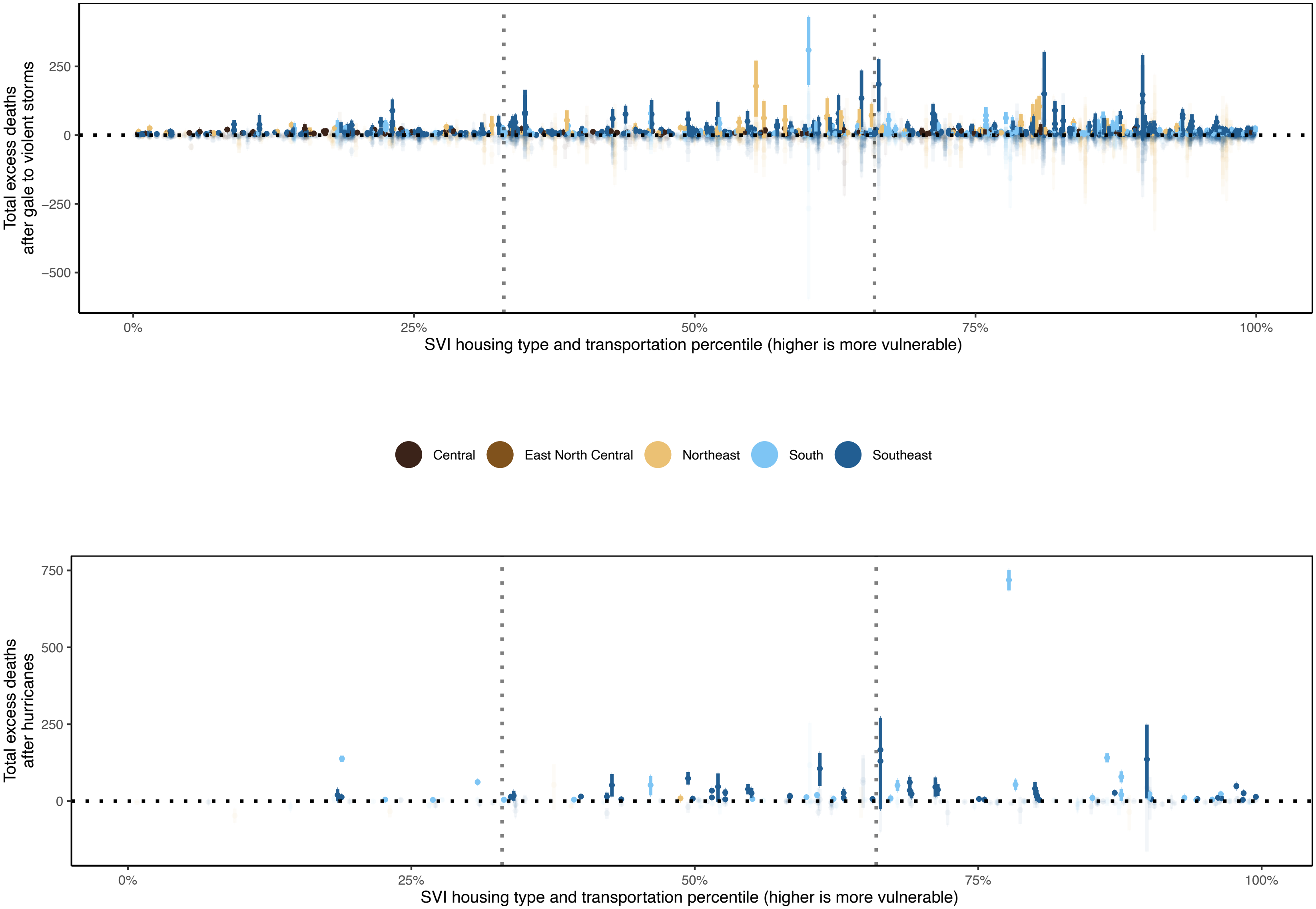

Fig. S5. Estimated annual county-level excess deaths after tropical cyclones categorized by gale to violent-force (top) and hurricane-force (bottom) events against housing type and transportation percentile Social Vulnerability Index (SVI) percentile, 1988–2019. Dots show the point estimates and whiskers represent 95% credible intervals. Vertical dotted lines represent boundaries of SVI tertiles. Highlighted points represent counties with a posterior probability>95% of excess deaths.

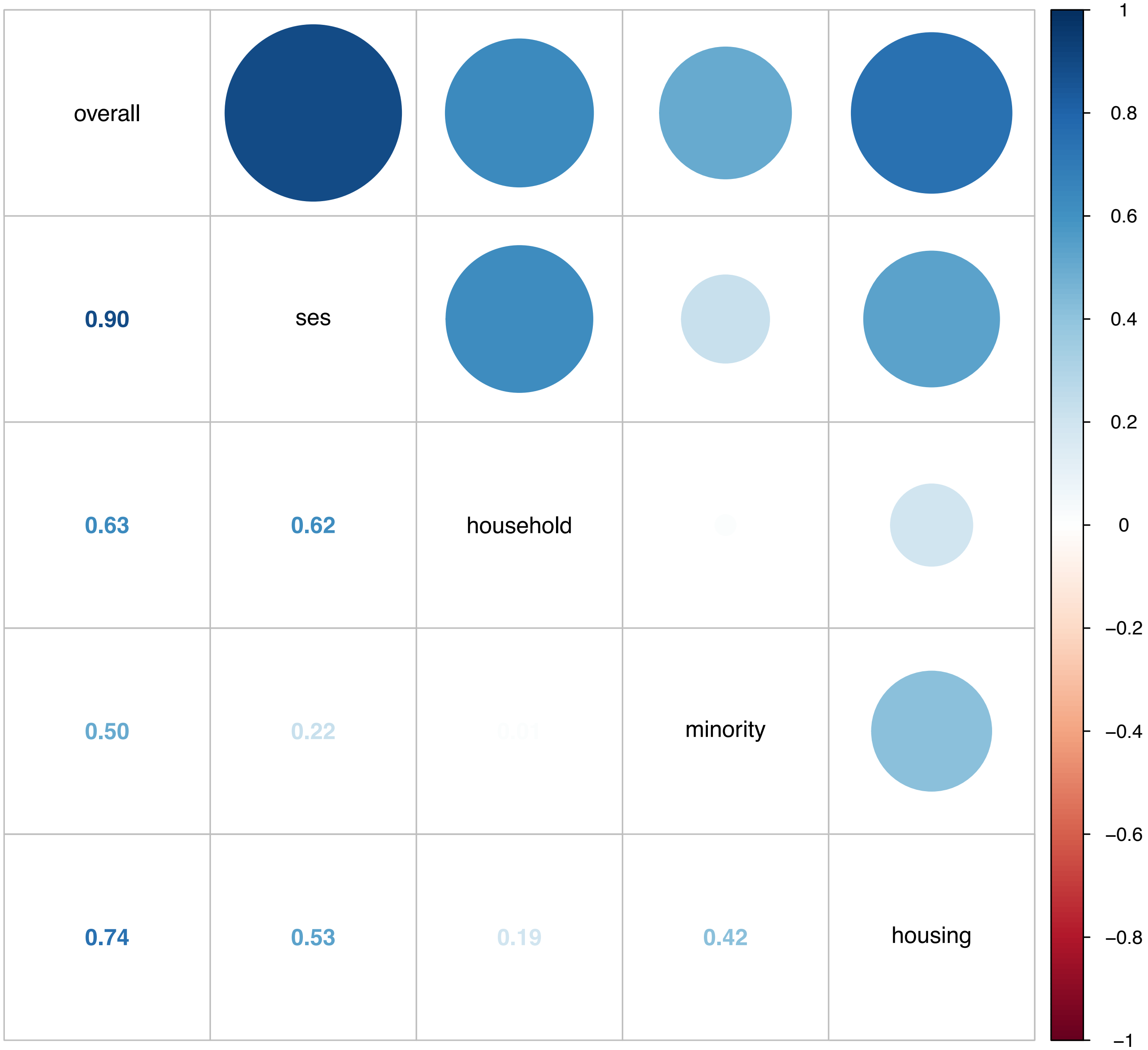

Fig. S6. Correlation plot of overall Social Vulnerability Index (SVI) and SVI components US-wide for 2018.

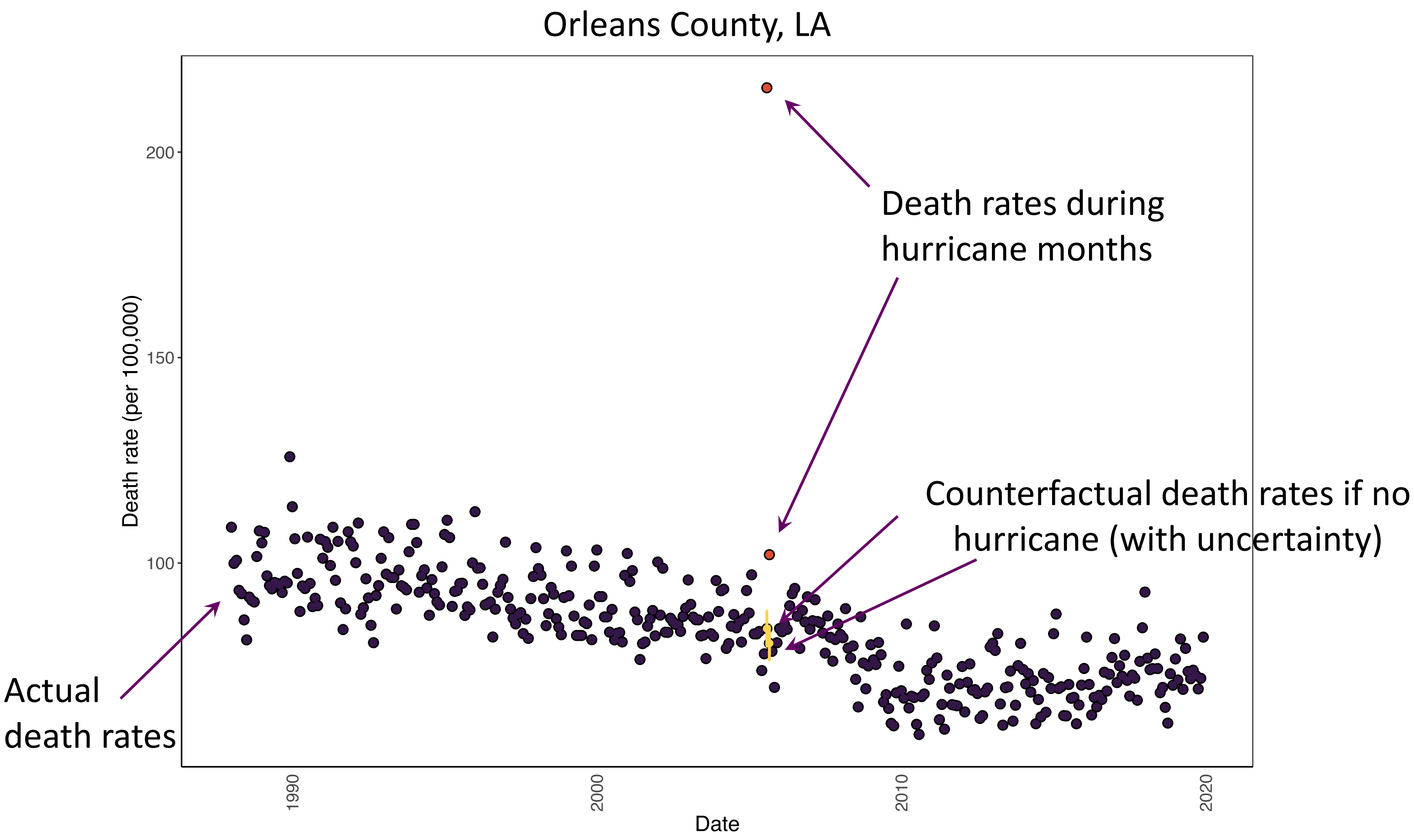

Fig. S7. Example of comparing actual death rates to counterfactual death rates in months with a hurricane in Orleans County, LA.

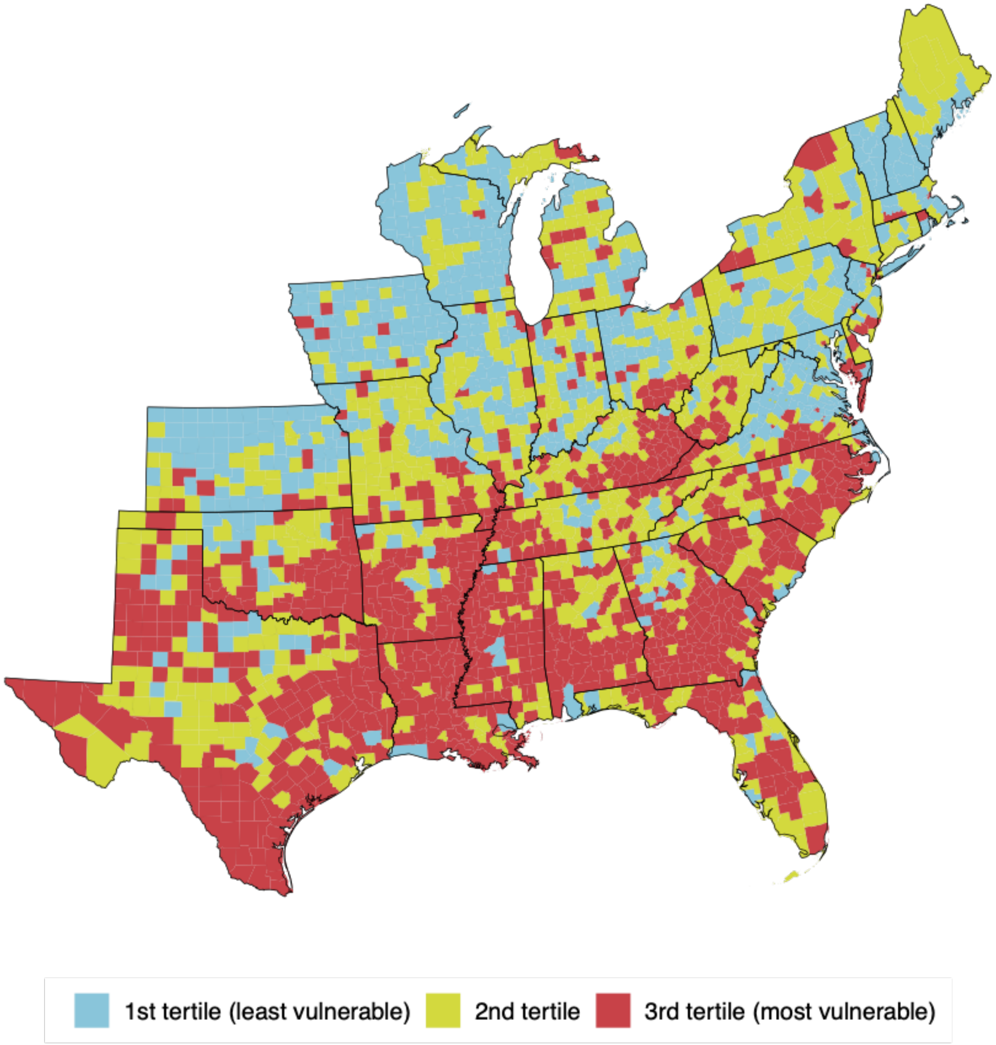

Fig. S8. 2018 Social Vulnerability Index (SVI) tertiles for 2018. The range is from 1 (least vulnerable) to 3 (most vulnerable).

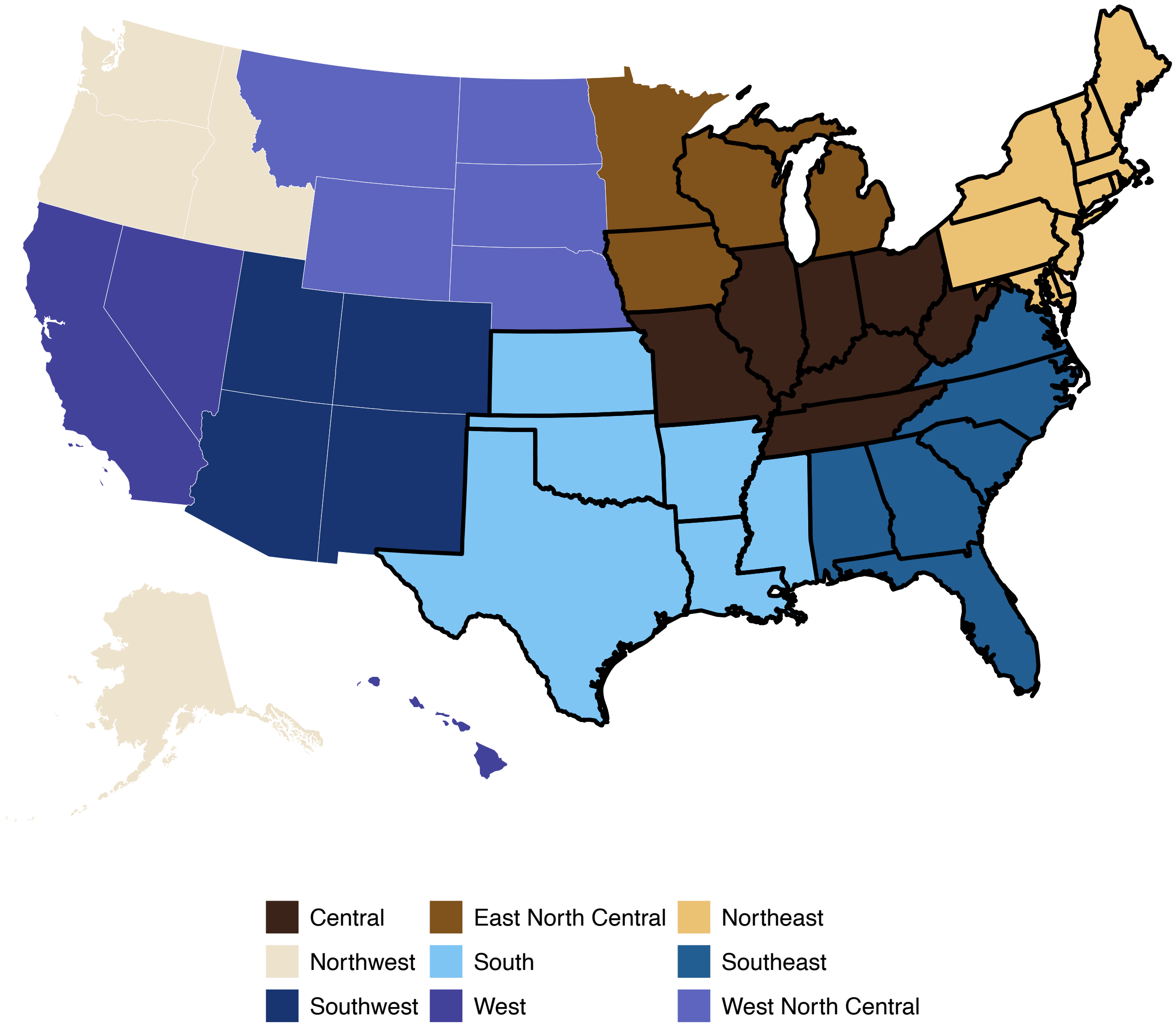

Fig. S9. Climate regions of the United States used by the National Oceanic and Atmospheric Administration. States included in our analysis are indicated with a black border.

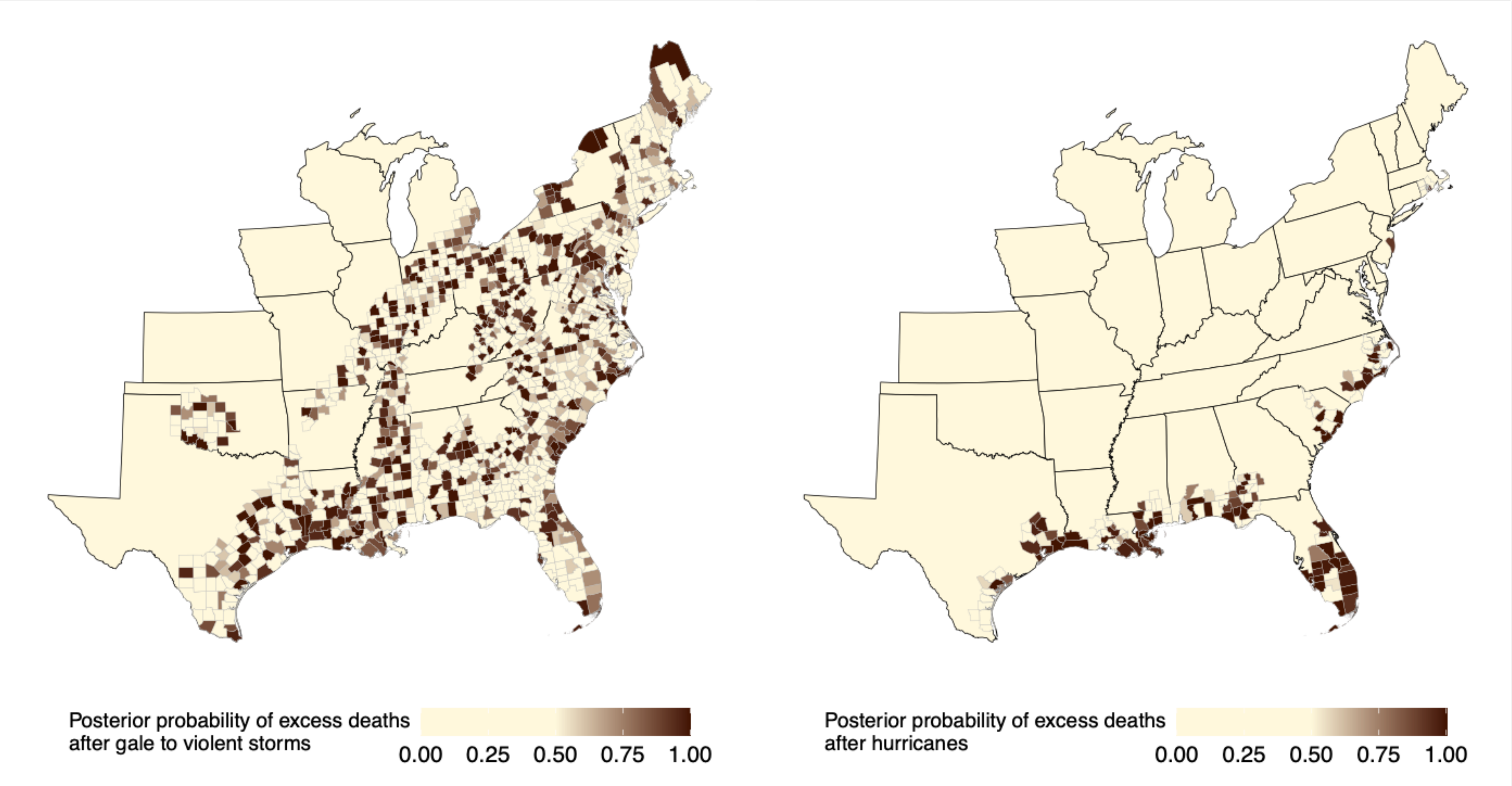

Fig. S10. Posterior probability of excess deaths >0 after tropical cyclones categorized by gale to violent-force and hurricane-force events by US county, 1988–2019.

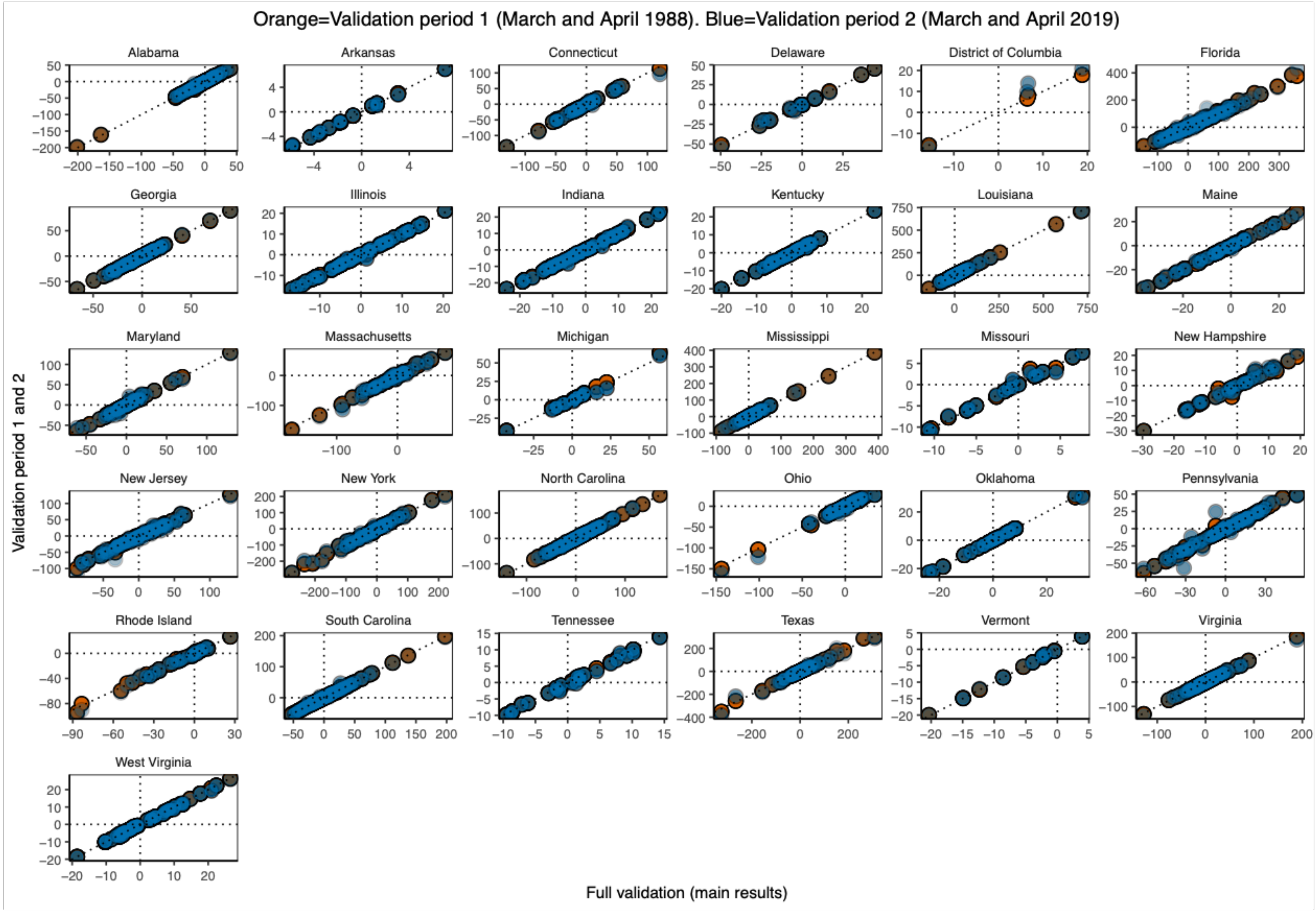

Fig. S11. Comparison of excess death estimates from the main analysis compared with sensitivity analyses using draws calculated only from validation period 1 (March and April 1988; in orange) or with using draws calculated only from validation period 2 (March and April 2019; in blue).

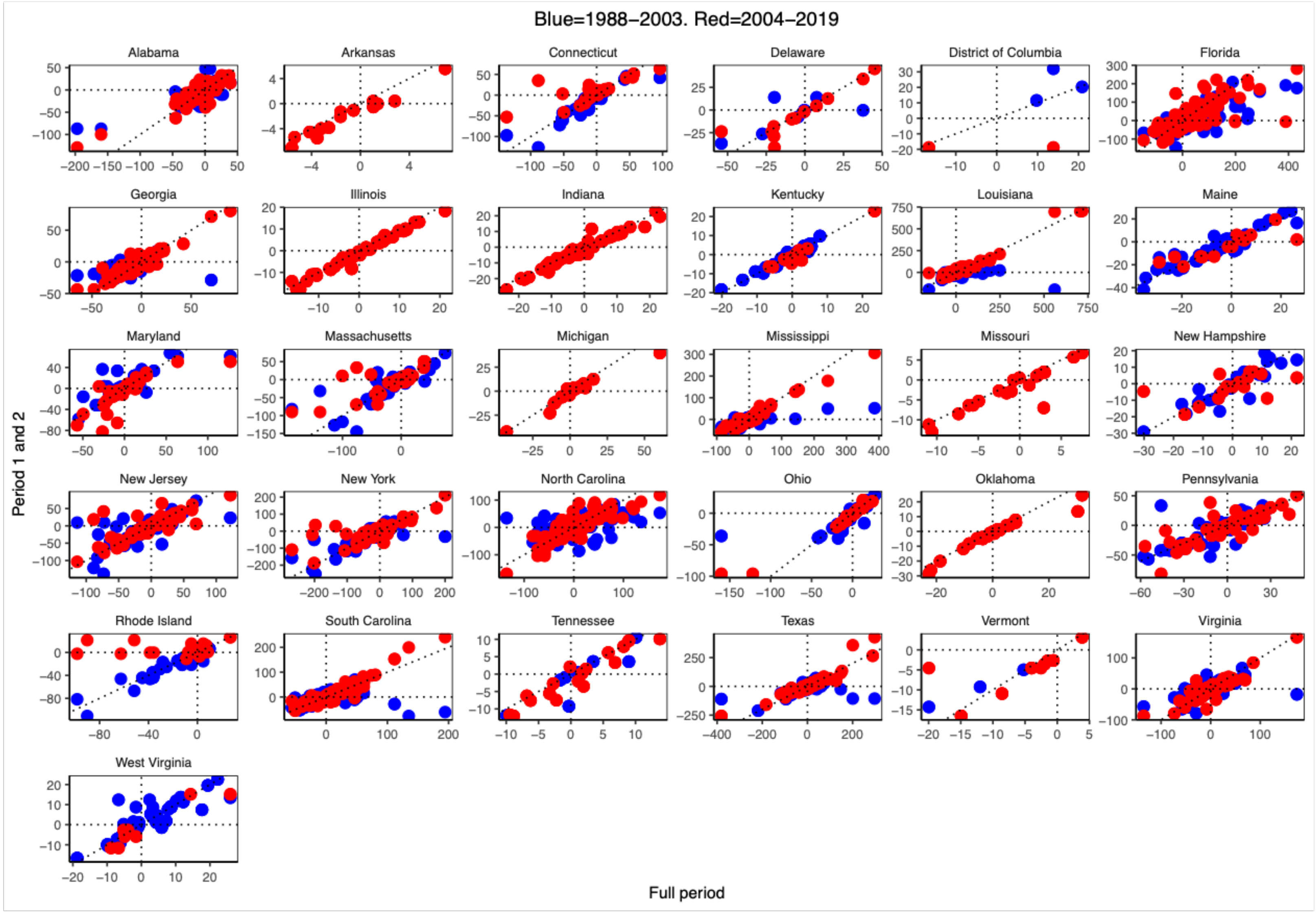

Fig. S12. Comparison of excess death estimates from the main analysis compared with sensitivity analyses only using data from the first half (1988–2003; in blue) or only using data from the second half (2004–2019; in red).

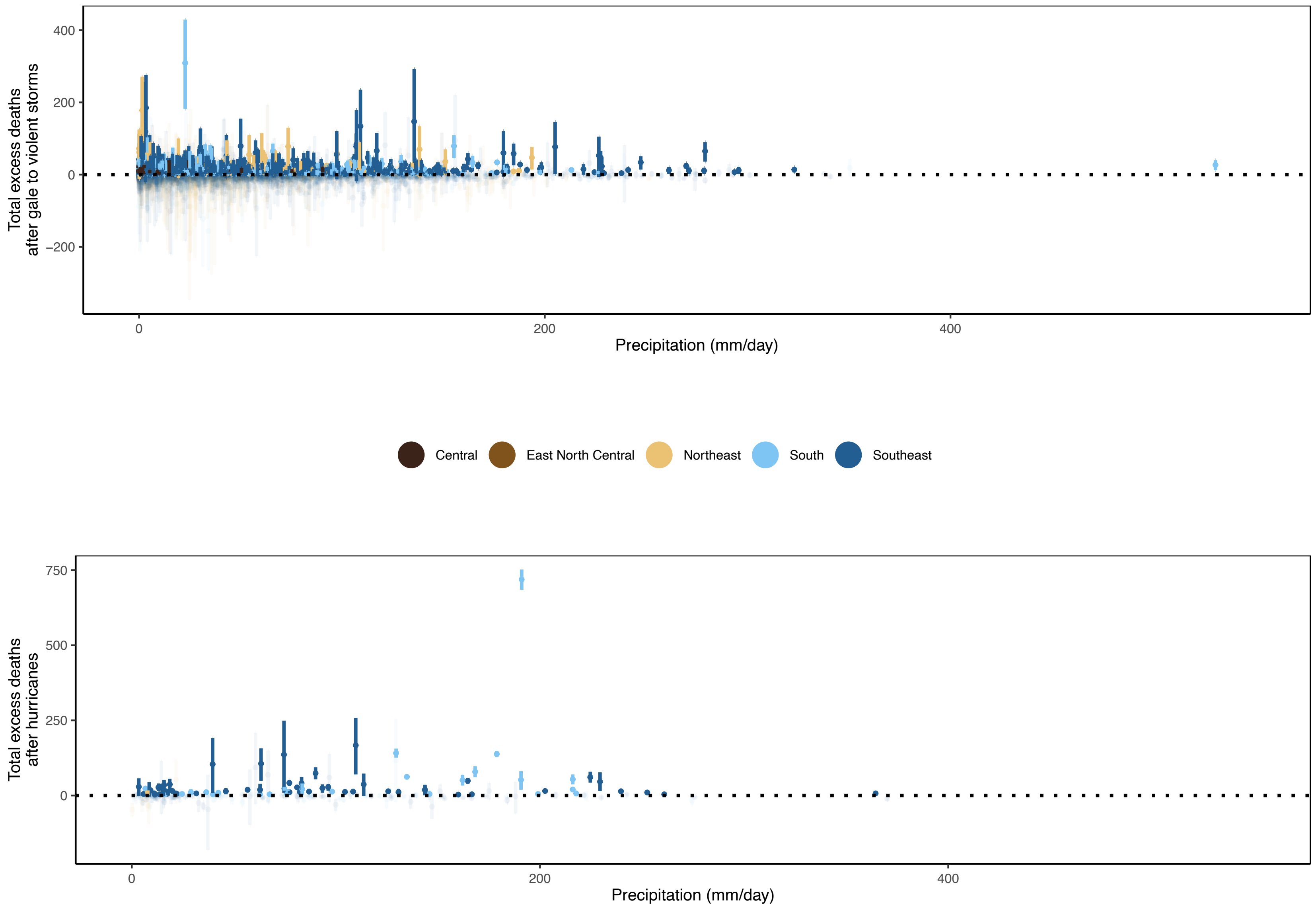

Fig. S13. **Estimated annual county-level excess deaths after tropical cyclones categorized by gale to violent-force (top) and hurricane-force (bottom) events against associated precipitation (mm/day), 1988–2019.** Dots show the point estimates and whiskers represent 95% credible intervals. Vertical dotted lines represent boundaries of SVI tertiles. Highlighted points represent counties with a posterior probability>95% of excess deaths.

Table S1. Estimated top twenty annual county excess deaths after tropical cyclone events, categorized by gale to violent- and hurricane-force events.

Rank	Gale to violent storm-force (≥34 knots and <64 knots)				Hurricane-force (≥64 knots)			
	Year	County	Estimated excess deaths	Posterior probability	Year	County	Estimated excess deaths	Posterior Probability*
1	2005	Harris County, TX	309 (182,429)	>99%	2005	Orleans Parish, LA	719 (685,752)	>99%
2	2016	Broward County, FL	185 (86,276)	>99%	1999	Broward County, FL	167 (70,258)	>99%
3	2012	Nassau County, NY	178 (67,271)	>99%	2005	Harrison County, MS	141 (126,156)	>99%
4	2017	Pinellas County, FL	150 (-10,303)	96.8%	2005	St. Bernard Parish, LA	138 (128,148)	>99%
5	2017	Miami-Dade County, FL	147 (2,292)	97.6%	1992	Miami-Dade County, FL	136 (9,249)	98.3%
6	2017	Palm Beach County, FL	134 (29,235)	98.9%	2005	Broward County, FL	130 (-26,271)	95.3%
7	2016	Miami-Dade County, FL	119 (-20,256)	95.5%	2008	Harris County, TX	117 (-22,255)	94.8%
8	2017	Hillsborough County, FL	112 (-29,244)	93.5%	2017	Lee County, FL	106 (49,157)	>99%
9	2012	Richmond County, NY	104 (56,143)	>99%	2005	Jefferson County, TX	79 (61,97)	>99%
10	1999	Miami-Dade County, FL	101 (-73,267)	88.0%	2004	St. Lucie County, FL	74 (54,94)	>99%
11	2016	Chatham County, GA	90 (55,125)	>99%	2005	Palm Beach County, FL	69 (-23,150)	94.5%
12	2019	Virginia Beach City, VA	89 (44,130)	>99%	2005	Hancock County, MS	62 (55,70)	>99%
13	2012	Kings County, NY	84 (-75,220)	85.1%	2004	Escambia County, FL	61 (43,79)	>99%
14	2016	Brevard County, FL	82 (-4,165)	96.7%	1999	Palm Beach County, FL	60 (-21,139)	92.6%
15	2017	Broward County, FL	81 (-20,173)	94.0%	2005	Calcasieu Parish, LA	54 (37,70)	>99%
16	2016	Marion County, FL	79 (10,145)	98.4%	2012	Ocean County, NJ	53 (-28,120)	91.3%
17	2012	Jefferson Parish, LA	79 (46,109)	>99%	2004	Sarasota County ,FL	52 (15,88)	>99%
18	1999	Essex County, MA	78 (20,130)	99.5%	2005	Jefferson Parish, LA	52 (19,81)	>99%
19	2019	Charleston County, SC	78 (41,114)	>99%	2008	Galveston County, TX	51 (33,69)	>99%
20	2017	Brevard County, FL	77 (1,146)	97.5%	2017	Monroe County, FL	49 (39,58)	>99%
*That excess deaths were > 0.								

Table S2. Total state-level excess deaths for four states most exposed to tropical cyclones throughout our study period, by Social Vulnerability Index (SVI) tertiles, using SVI percentiles for each county relative to the state it belongs, for counties with a posterior probability>95% of excess deaths.

**State**	**Category**	**SVI tertile 1**	**SVI tertile 2**	**SVI tertile 3**	**Posterior Probability SVIt3>SVIt1**	**Posterior Probability SVIt3>SVIt2**	**Posterior Probability SVIt2>SVIt1**
Florida	Gale to violent storm-force (≥34 knots and <64 knots)	490 (272,708)	1342 (866,1772)	1200 (749,1626)	>99%	14.4%	>99%
	Hurricane-force (≥64 knots)	104 (24,182)	835 (516,1109)	381 (225,524)	>99%	0.1%	>99%
Georgia	Gale to violent storm-force (≥34 knots and <64 knots)	29 (8,47)	50 (24,74)	491 (328,640)	>99%	>99%	96.1%
	Hurricane-force (≥64 knots)	Never exposed	Never exposed	15 (4,25)	Not applicable	Not applicable	Not applicable
Louisiana	Gale to violent storm-force (≥34 knots and <64 knots)	184 (118,242)	627 (446,792)	874 (661,1059)	>99%	>99%	>99%
	Hurricane-force (≥64 knots)	5 (2,7)	829 (731,917)	237 (188,282)	>99%	<1%	>99%
North Carolina	Gale to violent storm-force (≥34 knots and <64 knots)	352 (255,443)	441 (282,577)	1508 (1007,1945)	>99%	>99%	91.8%
	Hurricane-force (≥64 knots)	13 (6,19)	71 (32,97)	56 (20,88)	98.9%	17.9%	>99%

Table S3. Comparison of proportion of excess deaths in each SVI tertile, for overall SVI, as well as the four SVI constituent components.

Category	SVI tertile	SVI overall	SVI socioeconomic status	SVI household composition and disability	SVI minority status and language	SVI housing type and transportation
Gale to violent storm	1	17.0%	26.9%	36.5%	8.7%	16.5%
	2	33.3%	33.7%	31.4%	24.4%	35.7%
	3	49.6%	39.4%	32.2%	66.9%	47.9%
Hurricane	**1**	6.2%	10.2%	56.3%	2.5%	8.6%
	**2**	36.6%	38.9%	19.8%	9.9%	34.6%
	**3**	57.2%	50.9%	24.0%	87.6%	56.8%

Table S4. Combination of terms used in each of the 16 models for estimating number of monthly deaths that would be expected had the tropical cyclone not occurred. See Methods for an explanation of each term.

Model number	Global intercepts	Time slope	Non-linear (autoregressive) term	Seasonal term	Temperature anomaly terms	Validation mean % error	Number of draws taken
1	α_0		$\zeta_{month}^{(1)}$	$\theta_{month} + \omega_{month} * time$	$(\gamma + \nu_{month}) * temperature\ anomaly_{month}$	-0.07%	231
2	α_0		$\zeta_{month}^{(1)}$	$\theta_{month} + \omega_{month} * time$		0.17%	72
3	α_0		$\zeta_{month}^{(2)}$	$\theta_{month} + \omega_{month} * time$	$(\gamma + \nu_{month}) * temperature\ anomaly_{month}$	-0.28%	18
4	α_0		$\zeta_{month}^{(2)}$	$\theta_{month} + \omega_{month} * time$		-0.26%	25
5	α_0		$\zeta_{month}^{(4)}$	$\theta_{month} + \omega_{month} * time$	$(\gamma + \nu_{month}) * temperature\ anomaly_{month}$	-0.12%	118
6	α_0		$\zeta_{month}^{(4)}$	$\theta_{month} + \omega_{month} * time$		-0.37%	0
7	α_0		$\zeta_{month}^{(6)}$	$\theta_{month} + \omega_{month} * time$	$(\gamma + \nu_{month}) * temperature\ anomaly_{month}$	-0.26%	27
8	α_0		$\zeta_{month}^{(6)}$	$\theta_{month} + \omega_{month} * time$		-0.29%	17

9	α_0	$\beta * \text{time}$	$\zeta_{month}^{(1)}$	$\theta_{month} + \omega_{month} * time$	$(\gamma + \nu_{month}) * temperature\ anomaly_{month}$	0.09%	178
10	α_0	$\beta * \text{time}$	$\zeta_{month}^{(1)}$	$\theta_{month} + \omega_{month} * time$		0.30%	14
11	α_0	$\beta * \text{time}$	$\zeta_{month}^{(2)}$	$\theta_{month} + \omega_{month} * time$	$(\gamma + \nu_{month}) * temperature\ anomaly_{month}$	0.02%	1000
12	α_0	$\beta * \text{time}$	$\zeta_{month}^{(2)}$	$\theta_{month} + \omega_{month} * time$		0.14%	102
13	α_0	$\beta * \text{time}$	$\zeta_{month}^{(4)}$	$\theta_{month} + \omega_{month} * time$	$(\gamma + \nu_{month}) * temperature\ anomaly_{month}$	-0.08%	224
14	α_0	$\beta * \text{time}$	$\zeta_{month}^{(4)}$	$\theta_{month} + \omega_{month} * time$		0.27%	21
15	α_0	$\beta * \text{time}$	$\zeta_{month}^{(6)}$	$\theta_{month} + \omega_{month} * time$	$(\gamma + \nu_{month}) * temperature\ anomaly_{month}$	0.22%	39
16	α_0	$\beta * \text{time}$	$\zeta_{month}^{(6)}$	$\theta_{month} + \omega_{month} * time$		0.27%	22
